# Amelioration of Cancer Employing Chitosan, Its Derivatives, and Chitosan-Based Nanoparticles: Recent Updates

**DOI:** 10.3390/polym15132928

**Published:** 2023-07-01

**Authors:** Tarun Virmani, Girish Kumar, Ashwani Sharma, Kamla Pathak, Md Sayeed Akhtar, Obaid Afzal, Abdulmalik S. A. Altamimi

**Affiliations:** 1 School of Pharmaceutical Sciences, MVN University, Haryana 121105, India; tarun.virmani@mvn.edu.in (T.V.); girish.kumar@mvn.edu.in (G.K.); ashwani.pharmacy@mvn.edu.in (A.S.); 2 Faculty of Pharmacy, Uttar Pradesh University of Medical Sciences, Etawah 206001, India; 3 Department of Clinical Pharmacy, College of Pharmacy, King Khalid University, AlFara, Abha 62223, Saudi Arabia; mdhusain@kku.edu.sa; 4 Department of Pharmaceutical Chemistry, College of Pharmacy, Prince Sattam Bin Abdulaziz University, Al-Kharj 11942, Saudi Arabia; o.akram@psau.edu.sa (O.A.); as.altamimi@psau.edu.sa (A.S.A.A.)

**Keywords:** chitosan, modified chitosan, biopolymer, chitosan nanoparticles, cancer, patent, co-delivery

## Abstract

The limitations associated with the conventional treatment of cancer have necessitated the design and development of novel drug delivery systems based mainly on nanotechnology. These novel drug delivery systems include various kinds of nanoparticles, such as polymeric nanoparticles, solid lipid nanoparticles, nanostructured lipid carriers, hydrogels, and polymeric micelles. Among the various kinds of novel drug delivery systems, chitosan-based nanoparticles have attracted the attention of researchers to treat cancer. Chitosan is a polycationic polymer generated from chitin with various characteristics such as biocompatibility, biodegradability, non-toxicity, and mucoadhesiveness, making it an ideal polymer to fabricate drug delivery systems. However, chitosan is poorly soluble in water and soluble in acidic aqueous solutions. Furthermore, owing to the presence of reactive amino groups, chitosan can be chemically modified to improve its physiochemical properties. Chitosan and its modified derivatives can be employed to fabricate nanoparticles, which are used most frequently in the pharmaceutical sector due to their possession of various characteristics such as nanosize, appropriate pharmacokinetic and pharmacodynamic properties, non-immunogenicity, improved stability, and improved drug loading capacity. Furthermore, it is capable of delivering nucleic acids, chemotherapeutic medicines, and bioactives using modified chitosan. Chitosan and its modified derivative-based nanoparticles can be targeted to specific cancer sites via active and passive mechanisms. Based on chitosan drug delivery systems, many anticancer drugs now have better effectiveness, potency, cytotoxicity, or biocompatibility. The characteristics of chitosan and its chemically tailored derivatives, as well as their use in cancer therapy, will be examined in this review.

## 1. Introduction

Cancer is the most confounding illness of the 21st century and is growing enormously without any discernment based on cell type, age, or gender [1]. Although incredible advancements in cancer treatment have been achieved, the prevalence and mortality rates are still the highest across the world. The World Health Organization estimated that 10 million deaths in 2020 were credited to cancer, and 400,000 children are diagnosed with cancer annually. It is anticipated that there will be 29.5 million new cases and 16.4 million cancer-related deaths by 2040, which is a serious health burden around the globe [2]. An additional fact is that about one in six deaths is attributed to cancer across the globe. This exceedingly endemic disease is currently estimated to be the second most prevalent cause of mortality after cardiovascular diseases [3]. Approximately 70% of cancer deaths belong to low and middle-income nations, depicting a significant impact on low and middle-income nations both physically and economically [4]. However, making little lifestyle changes, abstaining from drinking, and quitting chewing tobacco can lower cancer cases by around 30% to 50% [5].

**Figure 1:**
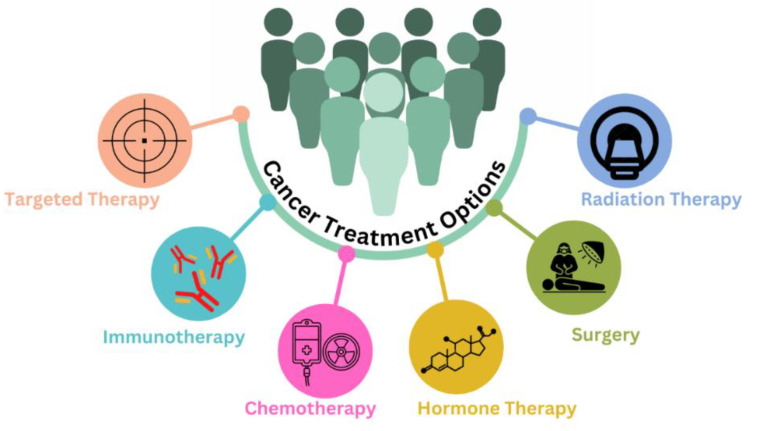
Various treatment modalities for the management of cancer.

The treatment of cancer is challenging and necessitates a combination of several parallel and subsequent therapies, such as surgery with chemotherapy and surgery with radiotherapy, depending on the type and stage of the cancer. Although chemotherapy is mainly employed to treat cancer, there are various alternative treatment options for cancer, as depicted in Figure 1.

The Food and Drug Administration (FDA) has permitted the use of more than 300 chemotherapeutic agents to treat cancer. But the heterogeneous nature of cancer has limited the efficacy of all these chemotherapeutic agents [6]. These agents encounter several difficulties, such as drug-related adverse effects, drug resistance, the insensitivity of cancer cells to treatments, and a lack of drug targeting [7,8]. In addition, more crucially, some of the key downsides of chemotherapeutic agents include the need for high doses, poor absorption, a low selectivity index, non-specific interactions, and patient discomfort [9,10]. Furthermore, chemotherapeutic agents demonstrate their effectiveness in treating cancer by specifically targeting cell proteins, nucleic acids, and carcinogenic signaling pathways that drive cancerous cells to adapt to changes in their environment and promote the spread of cancer [8]. Therefore, it is necessary to look for novel and advanced treatment alternatives for boosting the therapeutic efficiency of chemotherapeutic agents.

Advanced treatment options involve the nanotechnology approach, a rapidly evolving cutting-edge scientific area that integrates a variety of fields like chemistry, physics, and biology, as well as the creation of novel nano-dimension structures with therapeutic uses in pharmacology and the biomedical industry [11,12]. Numerous characteristics of the nanodimension, including optical, magnetic, and structural surface area ratios, make it an exciting subject for research in every way [13,14]. Due to the increased surface area of nanoscale medicines or devices, they serve as nanocarriers and nanoadsorbents and transport therapeutic substances, proteins, or probes [15,16]. These nanoscale methods comprise solid lipid nanoparticles (SLNs), liposomes, nanostructured lipid carriers (NLCs), nanoemulsions (NEs), polymeric nanoparticles (PNPs), polymeric micelles (PMs), carbon nanotubes (CNTs), and dendrimers [17,18]. Among the numerous kinds of nanocarriers, polymeric nanoparticles (PNPs) have been widely exploited for the delivery of anticancer drugs owing to various characteristics such as biodegradability, biocompatibility, reduced size, amplified surface volume ratio, and easier reformation of structure and surface [19] PNPs enable the protection of enveloping drug molecules and control or sustain the release of embedded drugs. The potential of PNPs to transport chemotherapeutic agents is unremittingly growing due to their capability to target the chemotherapeutic agents only on cancer cells [20]. Numerous polymers have been employed for the fabrication of PNPs, which include natural (cellulose, chitosan, gelatin, lysozyme, dextran, collagen, and albumin), synthetic (poly lactide-co-glycolide (PLGA), polylactic acid (PLA), thiolated poly methacrylic acid), and semisynthetic (methylcellulose) polymers [21].

**Figure 2:**
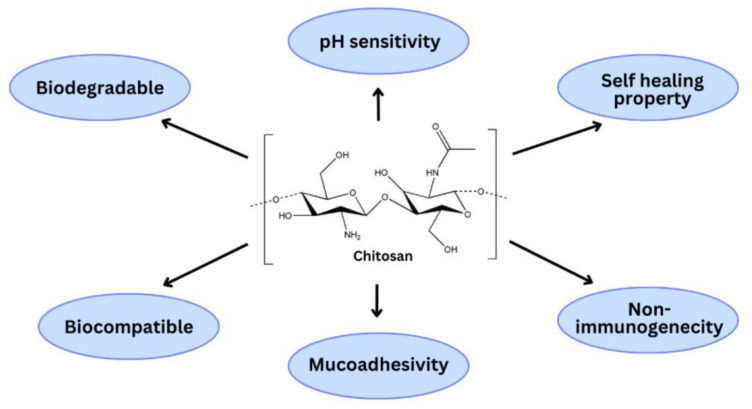
Chemical structure of chitosan along with model pharmaceutical properties.

Natural polymers have received a great deal of attention for the fabrication of PNPs for the delivery of anticancer drugs due to their possession of numerous characteristics: higher biocompatibility, biodegradability, interactions with biomolecules, controlled enzyme degradation, low immunogenicity, ease of surface modification, and economics [22,23]. Amongst these natural polymers, chitosan has received the courtesy of researchers as a drug delivery system to deliver chemotherapeutic agents due to distinct characteristics like great drug loading capability, sustained circulation capacity, multifunctionality, drug release at cancerous sites in an efficient manner, removal of cytotoxicity to non-cancerous cells, auspicious targeting, and permeability of cell membranes owing to the presence of a primary amine group in their chemical structure [24,25]. Chitosan provides specific targeting to cancerous cells due to functionalization with various specific polymers such as hyaluronic acid (HA), polyethylene glycol (PEG), folic acid (FA), RGD, etc. In a study, Almutairi FM et al. prepared hyaluronic acid-decorated chitosan nanoparticles loaded with raloxifene to treat lung cancer and found that raloxifene-hyaluronic acid-chitosan (RX-HA-CS) nanoparticles exhibited greater cytotoxicity against A549 cancer cells than raloxifene-hyaluronic acid (RX-HA) nanoparticles and raloxifene-chitosan (RX-CS) nanoparticles. This noteworthy dominance of A549 cell viability was attained through glucose uptake lessening, which resulted in reduced bioenergetics of cancer cells and stimulation of apoptosis through nitric oxide level elevation [26]. In another study, Ullah et al. prepared folic acid-decorated chitosan nanoparticles embedded with 5-fluorouracil and investigated whether cytotoxic potential was improved in the presence of folic acid. The folic acid-decorated chitosan nanoparticles showed greater cytotoxicity than plain, undecorated chitosan nanoparticles in colon cancer [27]. This depicts that functionalization of chitosan nanoparticles with specific molecules improves the site-specific potential of drug molecules embedded in them. The chemical structure of chitosan and its properties as an ideal drug delivery system are depicted in Figure 2.

## 2. Conventional Chemotherapeutic Treatment

Chemotherapy is perceived to be the most successful form of treatment for cancer since it specifically targets cancerous cells and kills them, preventing their spread or decreasing their growth [24]. Chemotherapy has a long history that dates back to the early 20th century, but its use to treat cancer was started in the 1930s. The word “chemotherapy” was created by the German scientist Paul Ehrlich to refer to the use of chemicals to cure the disease [28]. Ehrlich had a keen interest in alkylating agents. It was discovered that soldiers exposed to mustard gas during the First and Second World Wars had lower numbers of leukocytes. As a result, Gilman utilized nitrogen mustard as the first chemotherapeutic drug to treat lymphomas in 1943. Alkylating agents for the treatment of cancer, such as cyclophosphamide and chlorambucil, were created in the years that followed [29]. Thus, progress in chemotherapeutic agents ensued, and some anticancer drugs were developed. Various chemotherapeutic agents employed in the treatment of cancer have been summarized in Table 1.

Some of the drugs like vincristine, vinblastine, and paclitaxel listed in Table 1 are of plant origin, which has prompted scientists to search for more plant-derived drugs. Numerous studies have focused on plant-derived agents’ medicinal properties, which are now widely used for the treatment of various kinds of ailments, especially cancer [3]. The focus on plant-derived agents for the treatment of cancer is attributed to various characteristics such as low adverse effects, action via several paths, and low cost therapy [30,31]. Some plant-derived anticancer drugs having great anticancer potential include paclitaxel, beta-lapachone, lapachol, colchicine, curcumin, resveratrol, quercetin, naringin, allicin, piperine, epigallocatechin, andrographispaniculata, glinuslotoides, saussurealappa, punicagranatum, betulinic acid, and ashwagandha [8,32]. These plant-derived anticancer agents owe their anticancer properties to numerous mechanisms like the prevention of cancer cell-triggering proteins, enzymes such as topoisomerase, cyclooxygenase, CDK4 kinase, CDK2, Cdc2, MAPK/ERK, MMP, initiation of DNA repair mechanisms, signaling pathways, stimulation of antioxidant action, or prompting of the development of protective enzymes (Caspase-3, 7, 8, 9, 10, 12) [33].

## 3. Obstacles Associated with Conventional Chemotherapeutic Agents

The therapeutic efficacy of the chemotherapeutic agents available to treat cancer is limited for some reasons, including their non-specific nature, countless adverse effects, higher dose requirements, short half-lives, widespread biological distribution, drug resistance, drug instability, poor aqueous solubility, low bioavailability, and lack of drug targeting [34,35]. These chemotherapeutic agents are unable to differentiate between cancerous cells and non-cancerous cells, resulting in the destruction of normal cells and the initiation of serious toxic effects that are lethal [36]. Some of the frequently reported toxic effects include low blood counts, exhaustion, mouth discomfort, nausea, vomiting, loss of appetite, constipation or diarrhea, hair loss, skin changes or responses, pain or nerve alterations, and reduced fertility and sexuality [37]. Rarely, chronic use of conventional chemotherapeutic agents may cause the emergence of secondary cancer [24].

However, the extremely broad range of toxic effects reported strongly suggests that the foremost challenge in chemotherapeutic agent-based cancer treatment resides in the targeting stage of these agents, or more specifically, in the successful transport and distribution of these agents in the body [38]. They do not appear to get to the intended cancer cell, and they do not seem to get there intact either. As a result, a chemotherapeutic agent spreads freely throughout the body, which is why side effects occur. This is the fundamental reason why, even though various chemotherapeutic agents are quite effective at eliminating or killing cancer cells in laboratories, they have fallen short of demonstrating progressive improvement when used on humans [24]. Targeted drug delivery and controlled drug release thus stand out as one of the most significant and promising approaches to enhancing the effectiveness of chemotherapeutic agents, which in laboratory settings demonstrate substantial efficacy while significantly decreasing the toxic effects [39,40]. Hence, there are mainly two obstacles to the effective treatment of cancer using chemotherapeutic agents: the inability to stop the agent from interacting with healthy non-cancerous cells, especially when it is in free form, and the delivery of the agent to the tumor site properly. This has resulted in the development of novel drug carriers such as liposomes, SLNs, NLCs, polymeric micelles, NEs, nanosuspensions, quantum dots, CNTs, and dendrimers.

PNPs have emerged as crucial targets for delivering chemotherapeutic agents to cancerous cells owing to their unique attributes [41]. The trademark qualities of PNPs as cancer drug delivery systems, including controlled drug release, targeted drug delivery, increased therapeutic efficacy, and safety, have been demonstrated in numerous studies [42,43]. Due to the attachment of targeting ligands to the surface of PNPs, they are frequently used as drug delivery systems for chemotherapeutic agents. The controlled release of loaded chemotherapeutic agents is made possible by the surface modification of these PNPs with targeting ligands, which allows recognition by particular receptors or ligand binding sites that are overexpressed on cancer cells or at target areas [44,45]. In addition, to prevent the PNPs from being phagocytosed and opsonized by circulating phagocytic cells, hydrophilic chains might be added to their exterior. PNPs can be fabricated using synthetic (poly lactide-co-glycolide (PLGA), poly (ɛ-caprolactone) (PCL), polylactic acid (PLA), semi-synthetic (methylcellulose), and natural (lysozyme, gelatin, cellulose, dextran, chitosan, collagen, and albumin) polymers [8]. Natural polymers have received a great deal of attention for the fabrication of PNPs for the delivery of chemotherapeutic agents due to their higher biocompatibility, biodegradability, interactions with biomolecules, controlled enzyme degradation, low immunogenicity, ease of surface modification, and economics [22].

## 4. Chitosan and Its Derivatives for Anticancer Drug Delivery

Chitosan is a versatile polycationic, linear natural polysaccharide composed of N-acetyl-β-(1-4)-D-glucosamine and β-(1-4)-D-glucosamine units and originated from chitin employing the process of deacetylation [46]. Chitosan has drawn distinct attention as a drug delivery system because of its superior chemical and biological characteristics. Owing to its polycationic makeup, chitosan is a bioadhesive polymer that rapidly adheres to negatively charged surfaces like mucosal membranes. In this manner, it strengthens the adherence to the mucosal surfaces, thereby lengthening the duration that drug molecules are in contact with them [47]. The complex characteristics of chitosan facilitate the delivery of anionic drugs, low molecular weight drugs, and polyanionic compounds like SiRNA and DNA [48]. Chitosan possesses a positive charge, and cyclodextrin has a negative charge. The gelation of chitosan with cyclodextrin provides a modified chitosan drug carrier with the capability to carry hydrophilic as well as hydrophobic drugs. Cyclodextrin has a truncated cone structure with a hydrophilic outer surface and a hydrophobic interior cavity. As a result of hydrophobic interactions, a variety of lipophilic drugs can be loaded into the hydrophobic cavity of *β*-cyclodextrin, leading to improved solubility, loading efficiency, and stability of lipophilic drugs [49]. The drug-carrying capacity of chitosan increases with an increase in charge, which shows its ability to act as a pH-dependent drug carrier [50].

Chitosan also possesses permeation enhancement property which depends upon the positive charge of the polymer. Chitosan, having a high molecular weight and a great degree of deacetylation, provides improved epithelial permeability and hence causes the passage of polar drugs through epithelial surfaces [51]. It has better biocompatibility and poor toxicity. It is a biodegradable polymer that breaks down into harmless compounds that are fully absorbed by the body [52]. It also possesses efflux pump inhibitory properties due to which it blocks certain transporter proteins on the membrane of intestinal epithelial cells or enterocytes that release xenobiotics, primarily drugs, making these transporters one of the key elements of drug resistance mechanisms [53]. The presence of hydroxyl and amino groups in their structure provides prominent properties like pH sensitivity, gelation capability, improved permeability, and antimicrobial activity, which possess significant consequences for controlled drug release and targeted drug delivery [54,55]. These incredibly alluring qualities have increased interest in chitosan and its derivatives in recent years, leading to the development of safe and effective drug or gene carriers for cancer-targeted delivery systems.

**Figure 3:**
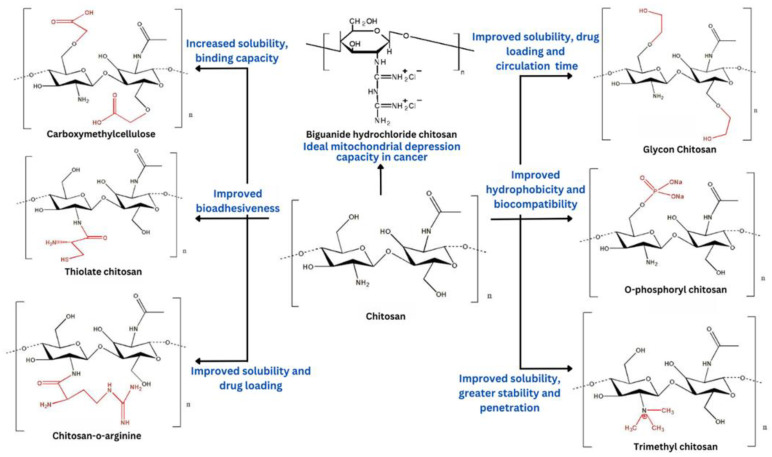
Modified chitosan along with their characteristics for improved drug delivery.

The properties of chitosan can be modified by employing physical and chemical modifications of the hydroxyl groups and free amino groups present in its structure [56]. Due to the presence of ridiculous functional groups, namely the amino group, primary hydroxyl, and secondary hydroxyl group at C-2, C-3, and C-6 positions, respectively, the parent structure of chitosan can be modified through acylation, alkylation, esterification, etc. to get modified chitosan derivatives having desirable physical, chemical, and biological characteristics, as depicted in Figure 3 [57].

By providing modifications in molecular weight, crosslinking, degree of deacetylation (DD), functional groups and moieties, synchronized anions or polyanions, etc., the modified chitosan has improved properties [50]. For instance, the solubility of chitosan can be significantly increased by adding tiny chemical groups like hydroxypropyl or carboxymethyl groups to its structure [58]. To introduce an anionic character with water-soluble properties, superior paste fluidity, a high water-reducing ratio, and anticoagulant properties, the cationic property can be reversed via sulfonation. With an increase in the crosslinking of chitosan, the mechanical strength of particles also increases [59]. Particles with a high degree of crosslinking exhibit decreased swelling, inner water infiltration, and outside drug diffusion. Drug release can be slowed by crosslinking, and burst releases can be avoided [60].

The mucoadhesive characteristic of chitosan can be enhanced by employing trimethylation of the primary amino group, PEGylation, or immobilization of the thiol groups present in its structure [61]. Amino groups in the structure of chitosan provide cationic properties, which are responsible for its solubility. This weak base is only soluble in diluted acidic solutions and insoluble in water and organic solvents because of the protonation of amine groups [62]. The higher amino group counts result in decreased molecular weight and improved solubility.

The viscosity of chitosan rises with a rise in molecular weight. Because hydrogen interactions across chains have a greater impact as molecular weight increases, solubility decreases [63]. The limited aqueous solubility restricts its application, whereas its solubility in acidic pH provides an opportunity to explore chitosan [64]. The reactive amine and hydroxyl functional groups of this biopolymer also render it vulnerable to chemical modification to enhance or acquire new features. To get around this obstacle, several derivatives of chitosan have been developed, which has multiplied its uses in the field of biomedicine for the delivery of active pharmaceutical ingredients by means of chemotherapy, immunotherapy, phytotherapy, and gene therapy [65]. At present, numerous drug delivery systems, namely microspheres, films, particles, nanofibers, nanocapsules, and nanocomposites, have been developed employing chitosan, which can be administered within the body via various routes like oral, intravenous, topical, nasal, and ocular routes [66].

Some immune checkpoints, such as Sting and CTLA-4, and programmed death-ligand 1 (PD-L1), are also affected by chitosan and its derivatives. Chitosan and its derivatives cause activation of the mitochondrial DNA-mediated cGAS-STING (Cyclic GMP-AMP synthase-Stimulator of Interferon Genes) pathway followed by the release of type I interferon, resulting in the immunostimulatory effect of chitosan [67]. Oral PD-L1 Binding Peptide 1 (OPBP-1), a proteolysis-resistant oral PDL-1 inhibitor that could specifically bind PD-L1 and disrupt its interaction with PD-1, was created using trimethyl chitosan. When loaded with trimethyl chitosan hydrogel, OPBP-1 demonstrated favorable oral bioavailability and a long half-life in rats and dramatically reduced tumor growth in mouse colorectal CT26 and melanoma B16-OVA models [68]. Cytotoxic T-lymphocyte antigen 4 (CTLA-4) molecules are one of the main barriers to priming T cells by dendritic cells (DCs). Therefore, it seems that blockade of such molecules facilitates T cell activation. In a study, suppression of the expression of the CTLA-4 molecule on tumor-infiltrating T cells by siRNA-loaded chitosan-lactate (CL) nanoparticles was facilitated [69].

## 5. Chitosan Nanoparticles as Anticancer Drug Delivery System

Considering the excellent characteristics of chitosan, the use of chitosan nanoparticles (CSNPs) has been exploited for the delivery of chemotherapeutic agents in the treatment of cancer. CSNPs provide various benefits, including ease of manufacturing, biocompatibility, biodegradability, mucoadhesiveness, improved permeation, economics, and ecofriendliness. The presence of cationic functional moieties and the electrostatic interaction of chitosan with various chemotherapeutic agents have provided it with excellent characteristics for efficient drug delivery in cancer [65]. Furthermore, CSNPs possess outstanding properties such as the ability to adjust the pharmacodynamic and pharmacokinetic of the drugs, the capability to transport both hydrophobic and hydrophilic drugs, controlled and sustained drug release, rapid penetration from biological membranes, increased accumulation of drugs in the internal environment of the diseased site, low immunogenicity, sustainability, and the capability of surface modification owing to the presence of amine and hydroxyl groups [70]. The transport of lipophilic drugs by CSNPs is possible by modification with specific molecules like cyclodextrin and hyaluronic acid. This modification of chitosan having a positive charge with hyaluronic acid or cylcodextrin having a negative charge via ionic interaction provides modified nanoparticles having the capability to carry lipophilic drugs [71]. In addition, CSNPs have good mechanical properties and thermal stability and can be modulated in different forms like powders, films, hydrogels, and porous frameworks [72]. CSNPs can also bypass the tight junctions present in epithelial cells [73]. The mucoadhesive property of CSNPs enables increased retention time at specific sites, enabling an increase in the therapeutic effectiveness of the drugs [74]. Based on their physicochemical characteristics, CSNPs employed for the loading of chemotherapeutic agents can be fabricated using a variety of techniques. The most practical and widely described methods include electrostatic adsorption, ionic gelation, covalent cross-linking, and self-assembly. Additionally, there are other methods for creating chitosan-based nanoparticles, including electrospinning, emulsification, and solvent evaporation [70,75].

**Figure 4:**
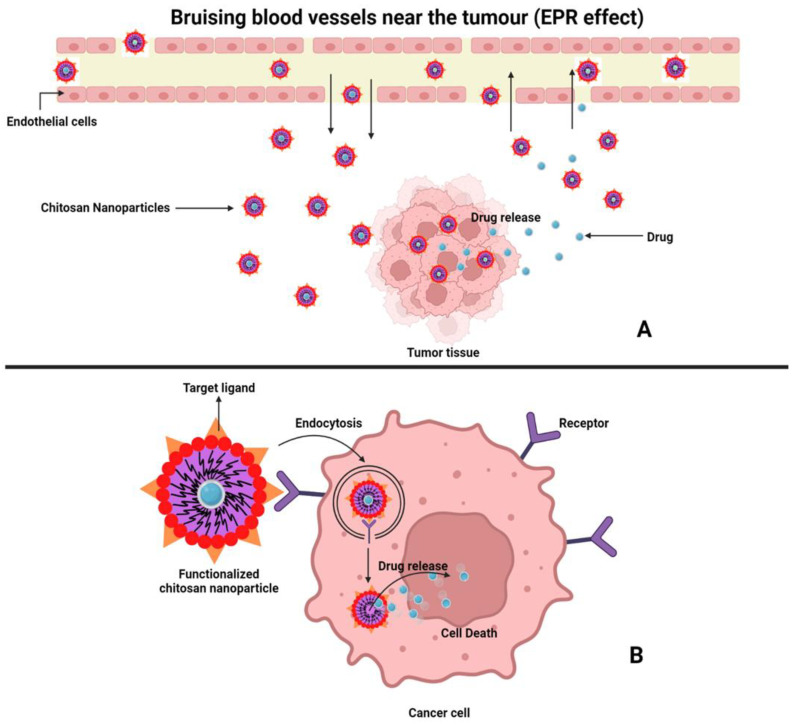
Representation of associated mechanisms for drug targeting to cancer site; Active targeting (**A**), Passive targeting (**B**).

To provide effective cancer drug therapy, it is necessary to target the chemotherapeutic agents at the cancer site. The drug targeting of cancer sites can be accompanied by two mechanisms, namely passive targeting and active targeting [76] as depicted in Figure 4.

In passive drug targeting, the CSNPs are transported to the cancer site using leaky tumor capillary penetrations. The enhanced permeability and retention (EPR) effect is then used to selectively transport drug-embedded nanoparticles into cancer cells. This is crucial for the uptake of CSNPs into cancer cells, especially in rapidly developing solid tumors. The ability of CSNPs to internalize into cancer cells can be increased by up to 50 times more than it is in normal cells [77]. These high ratios result from extensive angiogenesis with vascularization, irregular blood vessel structure between endothelial cells, damaged lymphatic drainage systems, rapidly proliferating tumor tissues, the absence of a smooth muscle layer in cancer blood vessels, the slow venous return of nanoparticles from the tumor environment, and the need for a higher oxygen supply to support rapid tumor proliferation [78].

CSNPs can potentially build up in cancer cells for a prolonged time owing to incomplete venous and lymphatic drainage. Furthermore, the mucoadhesive characteristics and the capacity to pass through momentarily open epithelial tight junctions might enhance the distribution of drugs across a variety of well-organized epithelia, including ophthalmic, oral, pulmonary, intestinal, etc [79]. However, several CSNP characteristics like size, shape, and surface characteristics may have a significant impact on the passive accumulation of nanoparticles in cancer regions because they have an impact on how nanoparticles are administered, distributed, metabolized, and eliminated. Because of the opsonic effect, CSNPs larger than 200 nm in diameter, non-globular in shape, and lacking biocompatible surfaces can be removed from human body serum with ease, while those smaller than 200 nm can significantly escape from the opsonic effect and offer a longer blood circulation time [80,81]. The uptake of nanoparticles in cells is influenced by their size and shape. Nanoparticles’ physical and chemical properties, such as size, shape, surface charge, and composition, influence how cells internalize them. Endocytic macrophages will expand to any size greater than 200 nm, aided by the addition of a ligand like folic acid or an epithelial protein. It is common knowledge that size is significant. In earlier investigations, smaller NPs were more likely to enter cells through endocytosis or diffusion, while larger NPs were more likely to enter cells through phagocytosis [82]. Surface modifications with different biomolecules, such as polyethene glycol, polylactic acid, and polylactic-co-glycolic acid, can significantly increase the physical stability and blood circulation time of CSNPs by evading the immune system and reticuloendothelial system [83]. The development and usage of metformin-modified chitosan could provide sensitization to the chemotherapy efficiency of cis-platinum due to its ideal capacity for selective mitochondrial accumulation, which further disrupted mitochondrial function. This, in turn, prevented the upregulation of programmed cell death ligand 1 (PD-L1) to prevent DNA damage repair in tumor cells, as well as decreased drug efflux and tumor metastasis [84]. Another chitosan derivative, biguanide-modified chitosan, had the greatest ability for mitochondrial depression, resulting in a reduction in the dosage required to interfere with mitochondrial function, reverse tumor hypoxia, and suppress MDR-1 expression [85]. Chitosan oligosaccharide (COS) might prevent the increased PD-L1 expression through the activation of AMPK and suppression of Signal Transducer and Activator of Transcription 1(STAT1) brought on by interferon (IFN) in a variety of tumors. Furthermore, COS itself dramatically inhibited CT26 tumor growth by promoting T cell infiltration in tumors. Additionally, we saw that adding COS to the common chemotherapy medication Gemcitabine (GEM) resulted in a more notable tumor remission [86].

Chitosan and its derivatives improve the sensitivity of other tumor therapies such as chemotherapy, radiotherapy, phototherapy, and chemoradiotherapy (CRT). Chitosan oligosaccharide may inhibit the abnormally up-regulated programmed cell death ligand 1 (PD-L1) after chemotherapy in various tumors via MAPK activation and STAT1 inhibition to improve the efficacy of T cell-mediated immune killing in tumors, suggesting that chitosan oligosaccharide may be used to enhance the effectiveness of current chemotherapies [86]. Chitosan nanoparticles have been shown to enhance the radiosensitivity of breast tumors in mouse models. Castro et al. demonstrated that combining chitosan/poly γ-glutamic acid nanoparticles (Ch/γ-PGA NPs) with radiotherapy induces antitumor immunity in the 4T1 orthotopic breast tumor mouse model [9]. Concomitant with glycated chitosan (GC), photodynamic therapy with Photofrin can improve the effects of inhibiting tumor growth, improving survival, enhancing local inflammatory reactions, and attracting acted immune cells to the tumor. It can also intuitively prove the systemic effect and long-term effect of laser immunotherapy (LIT) [87].

Chitosan may build up at the tumor site, starting the polarization of M1 macrophages and changing the immunosuppressive tumor milieu to an immune-supportive one, increasing the effectiveness of cancer immunotherapy and having an anticancer effect. In addition, chitosan has demonstrated potential as a drug carrier and therapeutic agent for its anticancer properties. Their ability to promote T-cell proliferation and hence start the production of cytokines is thought to be tied to their anticancer activity as a therapeutic agent. It has been noted that it blocks MMP-9 and strongly promotes apoptosis in tumor cells [88]. Dendritic cells (DCs) are activated by chitosan, which increases the anti-tumor activity of natural killer (NK) cells. Chitosan increased human NK cell IFN production when DCs were present. Chitosan worked by stimulating DCs to release pro-inflammatory cytokines like interleukin (IL)-12 and IL-15, which then stimulated NK cells’ STAT4 and NF-B signaling pathways, respectively. Additionally, chitosan increased NK cell cytotoxicity against leukemia cells and supported NK cell survival [89].

**Figure 5:**
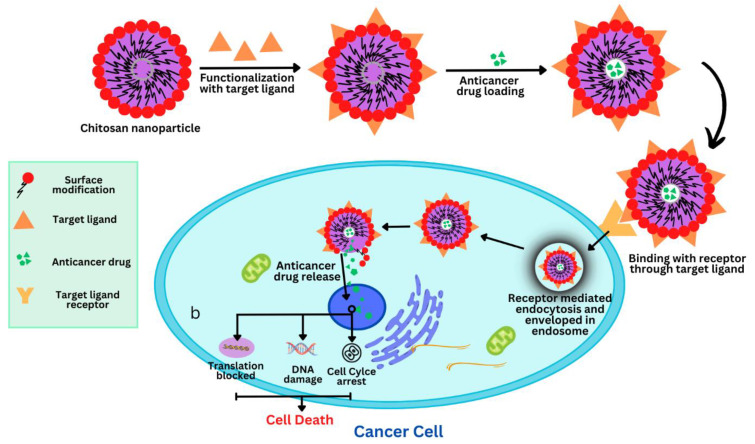
Representation of drug targeting to cancer cells employing chitosan-based nanoparticles.

The obstacles associated with passive drug targeting include the non-specificity of nanoparticles in locating target cells throughout the body, partly systemic side effects, and the cell membrane and cytoplasm of cancer cells that have several drug-capturing agents [70]. These obstacles can be avoided by employing drug targeting based on certain ligand-receptor methods and the physicochemical features of nanoparticles, which are termed “active drug targeting”. The principal targeting moieties involve folic acid, polysaccharides, nucleic acids, peptides, and proteins because they have a high affinity for attaching to their receptors [90]. Folic acid is being investigated as a potential ligand for the folate receptor (FR), which would allow it to target the surface of cancer cells and effectively endocytose nanoparticles [91]. The glycosylphosphatidylinositol-anchored membrane glycoproteins FR-α and FR-β are overexpressed on the surface of a variety of human cancer cells, including those from the breast, ovary, liver, kidney, lung, and others. Due to the high affinities of FR-α and FR-β, this ligand is a suitable molecule for receptor-mediated internalization of CSNPs [92]. Nucleic acids are a potential ligand due to certain benefits such as biostability, a distinctive three-dimensional structure, and a low dissociation rate. These benefits open the door for high binding affinity, biocompatibility, non-immunogenicity, non-toxicity, easy preparation, and cost effectiveness [93]. Using CSNPs, certain receptors that are overexpressed on the surface of different cancer cells have been used for peptide and antibody-based targeted delivery. For instance, integrins are a class of arginylglycylaspartic acid peptide (RGD) receptors that are abundantly expressed in a variety of cancers, including breast cancer. There are various types of RGDs, out of which cyclic peptides are more important for active drug targeting due to various characteristics such as tremendous binding discrimination, conformational stability, and low biodegradation [94]. A number of chemotherapeutic agents encapsulated in chitosan-based NPs have been summarized in Table 2. The drug targeting of cancer sites employing chitosan-based nanoparticles has been depicted in Figure 5.

Drug release from CSNPs depends mainly on two parameters: the composition of the drug product (drug, polymer, and excipients) and the method of fabrication. There are various mechanisms for drug release from the CSNPs, including diffusion-controlled release, dissolution-controlled release, degradation- or erosion-controlled release, and stimuli-triggered release. In the current scenario, stimulus-triggered release is extensively employed for the controlled release of the drug at a specific site for the treatment of numerous kinds of ailments. In stimuli-triggered release, drug release can be initiated using internal or external stimuli at a specific site to enable the selectivity of a drug at this site. Internal stimuli include intracellular or extracellular alterations in pH, temperature, enzyme activity, ATP, and hormonal activities, while external stimuli include light, ultrasound, magnetic fields, mechanical stress, etc. This mechanism for drug release provides drug delivery at the desired site with minimal adverse effects. Various stimuli that trigger chitosan-based nanocarriers have been summarized in Table 3.

## 6. Chitosan Nanoparticles (CSNPs) for Codelivery of Drugs in Cancer Treatment

Codelivery of chemotherapeutic agents has emerged as a crucial strategy to overcome the challenges associated with the administration of conventional chemotherapeutic agents and phytochemicals in monotherapy, leading to increased therapeutic efficacy in cancer and decreased side effects [133]. Codelivery of drugs in cancer is advantageous because it possesses a variety of characteristics, such as fewer doses, which increase patient compliance, a decrease in multiple drug resistance, and fewer dosages, which lessen side effects in non-cancerous cells. Additionally, several studies have demonstrated the benefits of combining chemotherapeutic medications with phytochemicals, including a synergistic anticancer impact, the reversal of multiple drug resistances, and a reduction in side effects [8]. Various chemotherapeutic agents have been codelivered employing CSNPs, resulting in improved therapeutic efficacy. Li et al. developed chitosan nanoparticles for the codelivery of 5-fluorouracil and leucovorin in the treatment of colorectal cancer. It was observed that both drugs exhibited burst release initially, followed by continuous release at a later stage. The drug release was initially affected by their drug concentration, which indicates that drug release from nanoparticles could be controlled by altering the drug concentration at the initial point [134]. Jia et al. fabricated mitomycin C and methotrexate-loaded PEGylated CSNPs for targeted drug delivery and synergistic effects. It was found that PEGylated CSNPs have nanometric size, constricted particle size, proper multiple drug loading, and sustained drug release. Based on in vitro cell viability testing, it was concluded that CSNPs showed concentration- and time-dependent cytotoxicity. Furthermore, it was observed that SCNPs could be taken effectively by cancer cells via folic acid receptor-mediated endocytosis [135]. In another study, Khan et al. fabricated CSNPs for the delivery of curcumin and cisplatin to treat ovarian cancer and observed that the optimized formulation exhibited a particle size of 225 nm and an encapsulation efficiency of 80%. The pattern of drug release displayed controlled drug release for both cisplatin and curcumin together and a lack of burst release. In addition, the cytotoxicity and uptake of both drugs in NPs showed augmented chemosensitization and increased therapeutic efficacy of both drugs [136]. Various chemotherapeutic agents codelivered employing chitosan nanoparticles have been summarized in Table 4.

## 7. Surface Modification of Nanocarriers Using Chitosan

Despite the wide applicability of various nanocarriers in the treatment of cancer, these have some issues that lead to their poor therapeutic efficiency. Liposomes suffer from various issues such as drug leakage during prolonged storage, poor stability in aqueous media causing reduced shelf life, destabilization of liposomal membranes by pH, bile salts, the acidic pH of the gastric region, and elimination from systemic circulation on intravenous administration. These issues cause a reduction in the efficiency of liposomes in delivering therapeutic agents at the desired site in sufficient amounts [145]. Lipid-based nanoparticles, namely solid lipid nanoparticles (SLNs) and nanostructured lipid carriers (NLCs), have poor stability in gastric pH on oral intake, poor encapsulation efficiency, poor stability, well-organized lipid structure, burst release of the drug, lack of drug targeting, and poor drug permeation, which cause obstacles in the therapeutic efficiency of drugs embedded in lipid nanoparticles [146,147]. Nanoemulsions (NEs) also have stability issues that lead to creaming, Ostwald ripening, sedimentation, coalescence, and flocculation, enabling poor therapeutic efficacy of drugs loaded in NEs [148]. In addition, PLGA nanoparticles suffer from poor residence time on mucosal tissues, resulting in poor efficiency [149]. To overcome all these issues, surface modification is a promising way that has been extensively used to improve the therapeutic efficiency of nanocarriers in recent years. Surface modification of nanocarriers can be accompanied by various approaches, such as ligands, polymers, fatty acids, and surfactants. Polymer-based surface modification is widely used to improve penetration capability, controlled drug release, drug targeting to a specific site, and drug payload capacity [50]. Hasan et al. fabricated curcumin-loaded liposomes, followed by their coating with chitosan. It was found that the addition of a chitosan layer greatly boosted the liposomal dispersion stability by reducing the hysteresis loop area, as seen by the increase/decrease stress ramp. After the addition of chitosan, the viscoelastic characteristics studied by small-amplitude oscillatory shear rheology showed an improvement in mechanical stability. The liposome membrane structure was not impacted by the chitosan layer or the encapsulated curcumin, according to small-angle X-ray scattering tests [150].

In another study, NLCs having chloroaluminiumphthalocyanine were fabricated and functionalized using chitosan. It was observed that the encapsulation efficiency of functionalized NLCs was 96%, whereas it was only 79% for non-functionalized NLCs. Both formulations showed significant cutaneous retention of embedded agents after 2 h and 4 h of the investigation, respectively. The PDT in BF16-F10 melanoma caused cell viability to decrease by 50% and 70% for functionalized NLCs and non-functionalized NLCs, respectively [151]. Various anticancer drug-loaded nanocarriers functionalized using chitosan have been summarized in Table 5, and patents related to chitosan-based NPs to deliver chemotherapeutic agents have been summarized in Table 6.

## 8. Conclusions

The prevalence and mortality of cancer have both been rising over the past few decades. Because of the sheer complexity of the disease, it has become extremely difficult, particularly concerning the quality of life of the patients. Recently, chemotherapy has been frequently used with surgery as a cancer treatment. However, chemotherapy has a low success rate as a treatment because of several issues, including side effects, drug resistance, the insensitivity of cancer cells to medications, a lack of targeting, and annoyance to the patient. Chitosan, a natural polycationic polymer derived from chitin, has advantageous properties such as biocompatibility, biodegradability, lack of toxicity, and mucoadhesiveness. It demonstrated promise in its application to cancer treatment through efficacious chemotherapeutic drug delivery. The presence of a reactive amino group on its backbone provides modified derivatives of chitosan with improved physiochemical and biological properties, which overcome the hurdle of poor aqueous solubility of chitosan. Chitosan and its modified derivatives can be employed to fabricate various drug delivery systems, including liposomes, nanoparticles, nanoemulsions, and metallic nanoparticles. CSNPs have emerged as interesting drug delivery systems for the delivery of chemotherapeutic agents in the treatment of cancer due to their possession of various advantages, including ease of manufacturing, biocompatibility, biodegradability, mucoadhesiveness, improved permeation, economics, and ecofriendliness. These nanoparticles can load both hydrophobic and hydrophilic drugs, shield the stability of drugs against various degradation mechanisms, and provide the release of drugs in a controlled and sustained manner at the target site. These have the potential to target the drugs using both active and passive mechanisms as well as stimuli responsiveness at cancer sites, which has centered the attention of researchers on using CSNPs for drug delivery in treating cancer. CSNPs have provided remarkable solutions to a variety of problems encountered while administering chemotherapeutic agents to the cancer site, and it is anticipated that they will continue to create new pathways for effective drug delivery for cancer treatment in the future. Moreover, despite huge progress in chitosan-based nanoparticles, a combination of chitosan with other biodegradable polymers to make novel drug carriers is unexplored, which can open the door for research in the future.

## Figures and Tables

**Table 1:** A compilation of traditional chemotherapeutic agents [8].

Category	Sub Class	Examples	Description
Alkylating agent	Nitrogen mustard	Cyclophosphamide, chlorambucil, bendamustine, ifosfamide, mechlorethamine, melphalan	Add an alkyl group to the guanine base of the DNA molecule, which causes inhibition of cross-linking of two strands, preventing replication of DNA overall and leading to cell death.
	Nitrosoureas	Carmustine, lomustine, streptozocin	
	Alkyl sulfonates	Busulfan	
	Triazines	Dacarbazine, temozolamide	
	Ethylenimines	Thiotepa, altretamine	
Antimetabolites	Folic acid antagonist	methotrexate	Cause inhibition of DNA synthesis by substituting the real metabolites that would be used in the regular metabolism
	Pyrimidine antagonist	5-fluorouracil, floxuridine, cytarabine, capecitabine, and gemcitabine	
	Purine antagonist	6-mercaptopurine and 6-thioguanine	
	Adenosine deaminase inhibitor	Cladribine, fludarabine, nelarabine, and pentostatin	
Platinum compounds		Cisplatin, carboplatin, oxaliplatin	Cause inhibition of DNA synthesis and transcription via binding of DNA to form interstrand and intrastrand cross-links.
Antimitotic agents	Vinca alkaloids	Vincristine, vinblastine	Cause inhibition of cell growth by causing a stoppage of the mitosis process.
	Texans	Paclitaxel, docetaxel, carbazitaxel	
Topoisomerase inhibitors	Topoisomerase–inhibitors	Topotecan, irinotecan	Cause inhibition of cell multiplying by stopping DNA replication, encouraging DNA damage, and tempting cell cycle arrest.
	Topoisomerase-II inhibitors	Etoposide	
Antibiotics	Anthracyclines	Daunorubicin doxorubicin, idarubicinepirubicin, valrubicin	Disrupt DNA via destroying topoisomerase, resulting in the arrest of the growth of cancer cells.
	Others	Mitoxantrone, bleomycin	
Hormonal drugs	Selective estrogen receptor modifier	Raloxifen, tamoxifen,	Cause the reduction in the speed of the cell cycle in MCF-7 cells, along with serving as proapoptotic factors.

**Table 2:** A collective data of chitosan-based NPs encapsulating chemotherapeutic agents.

Chitosan Based NPs	Characteristic of Chitosan or Modified Chitosan (MW, DD, and DS)	Encapsulated Chemotherapeutic Agent	Type of Cancer	Result Outcomes	Ref.
Chitosan	MW = 50,000–190,000 Da, DD = 75–85%	Curcumin	Triple negative breast cancer	Provided that concurrent delivery of tumor necrosis factor-related apoptosis-inducing ligand (TRAIL) expressing placental derived mesenchymal stem cells (PDMSCs) and curcumin nanoparticles efficiently brought about apoptosis in cancer cells, leading to significant inhibition of cancer growth.	[95]
Chitosan	MW = 50,000–190,000 Da	Mitomycin-C	Bladder cancer	Provided that CSNPs of mitomycin-C had 2.5 folds greater cancer inhibition effect.	[96]
Quaternized chitosan gallic acid-folic acid stabilized gold nanoparticles	MW = 500 kDa, DD = 81%	3,4,5-tribenzyloxybenzoic acid	Lung cancer	Displayed a 10-fold greater cytotoxicity than cisplatin in lung cancer cells and also depicted poor toxicity in normal cells.	[97]
Chitosan-pectinate	MW = 50,000–190,000 Da, DD = 75–85%	Curcumin	Colon cancer	Provided that NPs exposed to the different media had a retained matrix in an acidic environment and a distorted matrix in a pectinase-enriched environment.	[98]
Chitosan	DD = 75%	Paclitaxel	Triple negative breast cancer	Provided about 1.6 folds reduced IC_50_ for CSNPs than pure drugs, and hemolytic toxicity was 4 folds reduced than pure drugs.	[99]
Chitosan oligosaccharide-stabilized gold nanoparticles	MW = >10 kDa	Paclitaxel	Breast cancer	Showed great cytotoxic potential against MDA-MB-231 cells by inducing apoptosis with enhanced reactive oxygen species generation and changing mitochondrial membrane potential levels.	[100]
Chitosan	MW = 50,000–190,000 Da, DD = >75%	Cromolyn	Colorectal cancer	Provided that drug-loaded CSNPs applied an amplified protective anticancer effect via amending tumor pathology, cromolyn solution	[101]
Chitosan	MW = 50,000–190,000 Da, DD = >75%	Doxorubicin	Gastric cancer	Provided that the intestinal permeation of the drug was about 90% via loading in CSNPs.	[102]
PEGylated CSNPs labelled with monoclonal antibodies	-------	Doxorubicin	Breast cancer	Provided that anti-human epidermal growth factor-PEGylated doxorubicin-loaded CSNPs were most cytotoxic against MCF-7 cancer cells as compared to L-929 normal cells.	[103]
Chitosan	MW = 190–310 kDa, DD = 84.8%	Green tea essential oil	Liver cancer, breast cancer, colon cancer	Provided an extended blood circulation time of 4 h, 20.3 ± 2.1% localization of the encapsulating agent, and targeted cancer cells within 30 min.	[104]
RGD functionalized CSNPs	--------	Raloxifene	Breast cancer	Exhibited improved cellular uptake of CSNPs in α_v_β_3_ integrin-expressing breast cancer cells, induced greater cellular apoptosis, and inhibited breast cancer growth without producing any adverse effect on normal cells.	[105]
Self-assembled lecithin CSNPs	MW = 10,000–300,000 Da	Raloxifene		Provided 4.2 folds improved oral bioavailability of prepared NPs and decreased excretion of the drug in stools due to increased mucoadhesion.	[106]
Hyaluronic acid-CSNPs	MW = 35 kDa, DD = 92%	SiRNA	Lung cancer	Hyaluronic acid-decorated CSNPs directly delivered larger quantities of Cy3-labeled siRNA to the tumor sites, causing inhibition of tumor growth by downregulating *BCL2*, as compared to unmodified NPs loaded with siRNA	[107]
Chitosan	DD = 80%	Docetaxel	Breast cancer	Exhibited an improvement of 20% inhibition of growth of MDA-MB-231 cell lines compared to that caused by the free drug.	[108]
Chitosan	----------	Docetaxel	Breast cancer	Provided the reduced levels of BAX and BCL-2 gene expression in CSNP-treated cells compared to intact cells, while the BAX/BCL-2 ratio was ominously increased compared to the free drug.	[109]
Folic acid and poly (sulfobetaine methacrylate) CSNPs	MW = 50,000 Da. DD = 95.2%	Etoposide	Cervical cancer	Provided a strong inhibitory effect on the viability of HeLa cells. On intravenous administration to HeLa tumor-bearing mice, folic acid-conjugated NPs collected more quickly at the cancer site.	[110]
Fucoidan/Chitosan NPs	MW = 19,000–310,000 Da	Methotrexate	Skin cancer	Provided improved skin permeation and a significant decrease in pro-inflammatory cytokines formed by activated human monocytes.	[111]
Chitosan	MW = 50,000–190,000 Da, DD = >75%	Methotrexate	Breast cancer	Provided that radiolabeled drug-loaded CSNPs were highly uptaken in the cancer cell lines.	[112]
CSNPs & CSNPs modified with rituximab	MW = 19,000–310,000 Da, DD = 75%	Cisplatin	Breast cancer	Provided that drug-encapsulated CSNPs were more efficacious as anticancer treatment than CSNPs modified using rituximab due to a lack of specificity.	[113]
Chitosan	DD = 86%	5-Fluorouracil	Gastric cancer	Improved AUC _(0−t)_, MRT _(0−t)_ and *t*_1/2z_ of drug loaded in CSNPs were found than a solution of 5-fluorouracil.	[114]
Chitosan	---------	5-Fluorouracil	Skin cancer	Provided improved inhibitory effect on cancer cell lines than drug solutions.	[115]
Folic acid-coated CSNPs	MW = 310,000–375,000, DD = 83%	5-Fluorouracil	Colon cancer	Provided improved cytotoxicity of the NPs on coating with folic acid than uncoated CSNPs.	[27]
Chitosan	-------------	Chlorambucil	Breast cancer	Displayed retained anticancer effect along with low toxic effects. Additionally, it provided better cellular uptake into the MCF-7 cells.	[116]
Chitosan coated alginate	-----------	Cisplatin	Cervical cancer	Provided that chitosan-coated microparticles were more cytotoxic than uncoated microparticles and cisplatin solution. The chitosan-coated microparticles were more permeable than the uncoated microparticles.	[117]
Hydroxyapatite coated with CSNPs	DD = 90%	Curcumin	Brain cancer	Provided that both hydroxyapatite and chitosan potentiated the targeting efficiency of curcumin.	[118]
Hyaluronic acid coated CSNPs	50–160 kDa, DD = 85%	Tamoxifen	Breast cancer	Provided that hyaluronic acid-coated CSNPs were more cytotoxic than uncoated CSNPs.	[119]
Chitosan-hyaluronan	MW = 50,000–190,000, DD = 75–85%	Vinblastine sulfate	Leukemia	Revealed a rapid internalization of labeled chitosan/hyaluronan NPs on K-562 human myeloid leukemia cells	[120]

**Table 3:** A list of stimuli that trigger chitosan based nanocarriers for cancer treatment.

Type of Stimuli	Nanocarrier	Active Drug	Result Outcomes	Ref.
pH-sensitive	BSA-stabilized graphene (BSG)/chitosan nanocomposites	Doxorubicin	Provided that the release of drug from the nanocarrier had a noteworthy effect on the destruction of cancer cells at an acidic pH.	[121]
pH-sensitive	Chitosan oligosaccharides NPs	Doxorubicin	Exhibited precise pH responsiveness, depicting improved cytotoxicity.	[122]
pH-sensitive	PEGylated and poloxamer modified CSNPs	Doxorubicin	Provided improved ability of doxorubicin loaded in CSNPs to inhibit HeLa cancer cells.	[123]
pH-sensitive	Copper oxide NPs	Doxorubicin	Displayed adaptable nature of doxorubicin release at varying pHs.	[124]
Thermo sensitive	Chitosan-based polymeric hybrids	Curcumin	Provided that the thermoresponsive performance of each chitosan-based polymeric hybrid nanogel formulation was different owing to the grafted Pnipam length and density.	[125]
Redox responsive	Chitosan-based glycolipid nanocarrier	Paclitaxel	Provided a notable prevention of cancer growth with a rather low dose of paclitaxel.	[126]
Redox responsive	Chitosan micelles	Gambogic acid	Provided the greatest apoptosis stimulation and cytotoxicity than the non-sensitive and uncoated controls for the A549 NSCLC model.	[127]
Temperature responsive	Chitosan-*graft*-poly (*N*-isopropyl acrylamide) and sodium alginate-*graft*-poly(*N*-isopropyl acrylamide)	5-Fluorouracil	At a temperature above 37 °C, the hydrophilic to hydrophobic transition of poly(*N*-isopropylacrylamide) happened to cause an accumulation of NPs.	[128]
pH-responsive	Chitosan coated liposomes	Doxorubicin	Exhibited improved cellular uptake and enhanced therapeutic efficiency of the drug due to its Ph-dependent triggering.	[129]
Magnetic responsive	Palmitoyl chitosan NPs	Paclitaxel	Provided that the pattern of drug release can be controlled using a magnetic field.	[130]
Magnetic responsive	Chitosan nanocapsules	Coumarin 6	Exhibited a spherical morphology along with outstanding magnetic responsiveness.	[131]
pH and light responsive	Polymeric micelles	Camptothecin	Provided enhanced drug release under simultaneous triggering by PH and UV light, resulting in improved cytotoxicity.	[132]

**Table 4:** A collective database of Codelivered chemotherapeutic agents using chitosan-based NPs.

Chitosan/ Modified Chitosan	Characteristics of Chitosan or Modified Cgitosan (MW, DD, DS)	Codelivered Drugs	Type of Cancer	Average Particle Size	Result Outcomes	Ref.
Chitosan	MW = 100,000, DD = 85%	5-fluorouracil and leucovorin	Colorectal cancer	60 nm	Both 5-fluorouracil and leucovorin had burst releases initially, followed by constant and continuous releases later.	[134]
Hyaluronic acid-chitosan NPs	MW = 110 kDa	MiR-34a & doxorubicin	Triple negative breast cancer	185 nm	Exhibited improved anticancer effects of doxorubicin by conquering the expression of non-pump resistance as well as the anti-apoptosis proto-oncogene Bcl-2.	[137]
Chitosan	MW = 50 kDa, DD = 90%	IR780 & 5-Aminolevulinic acid	colon cancer	180 nm	Provided that drug-encapsulated CSNPs were stable in gastric pH, leading to improvements in oral absorption and deposition in CT-26 cells on oral administration.	[138]
Thiolated CSNPs	MW = 100–150 kDa, DD = 80%, DS = 60%	Curcumin & 5-Fluorouracil	Colon cancer	150 nm	Provided 2.5–3 fold greater anticancer effect and improved drug plasma concentration.	[139]
Carboxymethyl dextran CSNPs	MW = 400 kDa,	SiRNA & doxorubicin	Colorectal cancer	172 nm	Established that treatment with dual therapy in the form of NPs causes significant changes in EMT genes, apoptosis cell death, and inhibition of HCT-116 cells.	[140]
Chitosan	MW = 50,000–190,000 Da, DD = more than 90%	Raloxifene& doxorubicin	Breast cancer	25–35 nm	Provided 60% cancer cell inhibition, as illustrated by the XTT assay on MCF-7 cell lines.	[141]
Carboxymethyl chitosan	MW = 33,400 Da, DS = More than 80%	Curcumin & docetaxel	Lung cancer	190 nm	Improved in vitro and in vivo anticancer effects compared to docetaxel monotherapy and other nanocarriers embedded with docetaxel and curcumin alone.	[142]
PEGylated chitosan NPs	MW = 50,000–190,000 Da, DD = 75–85%	Ascorbic acid &oxaliplatin	Breast cancer	176 nm	PEGylation improved the apoptotic effect of ascorbic acid and oxaliplatin alone and increased the apoptotic effect of both drugs in combination on MCF-7 cells.	[143]
Folate targeted CSNPs	MW = 50,000 Da	METHFR shRNA/5-fluorouracil	Gastric cancer	83.2 nm	Provided enhanced therapeutic effectiveness of 5-fluororuracil and METHFR shRNA in code delivery compared to 5-fluororuracil and METHFR shRNA in solution.	[144]
Lipid chitosan hybrid NPs	MW = 50,000–190,000 Da, DD = 75–85%	Curcumin & cisplatin	Ovarian cancer	225 nm	The cytotoxicity and uptake of both drugs in NPs showed augmented chemosensitization and increased therapeutic efficacy of both drugs.	[136]

**Table 5:** Summarized list of anticancer drug loaded nanocarriers functionalized using chitosan.

Type of Nanocarrier	Therapeutic Moiety	Type of Cancer	Average Size	Result Outcomes	Ref.
Liposomes	5-Fluorouracil	Colorectal cancer	212–271 nm	Provided a delayed release of a drug over non-functionalized liposomes and a simple drug solution. exhibited more efficacy in killing cancer cells than non-functionalized liposomes and simple drug solutions.	[152]
Liposomes	Oleanolic acid	Breast cancer	167.44 nm	Exhibited a stronger anticancer effect than the simple solution of drug and uncoated liposomes.	[153]
Nanoliposomes	Curcumin	Breast cancer	120 nm	Improved encapsulation efficiency of 88%, compared to 65% for uncoated nanoliposomes, and exhibited a stronger inhibitory action on breast cancer cells.	[154]
SLNs	Docetaxel	Breast cancer	143–225 nm	Showed a slower drug release of docetaxel than uncoated SLNs. Also provided greater in vitro cytotoxicity and tumor-inhibiting ability than uncoated SLNs.	[155]
SLNs	Irinotecan	Colon cancer	430.77 nm	Displayed improved concentrations of irinotecan in the colon from chitosan-modified SLNs.	[156]
SLNs	Cisplatin	Cervical cancer	190 nm	Exhibited the minimal IC_50_ value of 0.6125 μg/mL for chitosan-coated SLNs, which was 1.156 μg/mL for uncoated SLNs. It also caused ominously greater apoptosis in cancer cells than uncoated SLNs.	[157]
NLCs	Chloroaluminiumphthalocyanine	Skin cancer	231.5 nm	Improved encapsulation efficiency of 96%, which was 79% for non-functionalized NLCs	[151]
NLCs	Tetrahydrocurcumin	Breast cancer	244 nm	Displayed ominous improvements in cell uptake, in vitro skin permeation, and noteworthy cytotoxicity to MD-MBA-231 breast cancer cells compared to uncoated NLCs of the same drug.	[158]
PLGA nanoparticles	Paclitaxel		132.8 nm	Improved cellular uptake of chitosan-modified PLGA nanoparticles than simple PLGA nanoparticles, whereas cell viability was decreased.	[159]
PLGA nanoparticles	Paclitaxel	Lung cancer	200–300 nm	Enhanced distribution index of a drug in the lungs.	[160]
PLGA nanoparticles	Tannic Acid/Vitamin E	Colon cancer	140–165 nm	Enhanced content of tannic acid/vitamin E from chitosan functionalized PLGA NPs than simple PLGA NPs and drug solutions.	[161]
PLGA/PCL NPs	Docetaxel	Colon cancer	177.1–287.6 nm	Enhancement in drug cytotoxicity by chitosan-decorated PLGA NPs than undecorated PLGA NPs and drug solutions.	[162]
PLGA nanoparticles	5-Fluorouracil	Colorectal cancer	188.1–302.2 nm	Displayed significant prevention of HT-29 cells compared to other NPs and drug solutions.	[163]
PLGA nanoparticles	Daunorubicin	Breast cancer	198.3 nm	Provided 10 times greater oral bioavailability than uncoated drug-loaded CSNPs.	[164]
Nanoemulsions	Piplartine	Skin cancer	96–112 nm	Exhibited 2.8-fold greater cytotoxicity of piplartine in chitosan-coated NE than drug solution against melanoma cells.	[165]
Gold NPs	Doxorubicin	Breast cancer	18 nm	Exhibited enhanced chemo-radiotherapeutic effect by reducing the viable cancer cells.	[166]
Magnetic NPs	Oxaliplatin & irinotecan	Colorectal cancer		Provided improved cytotoxicity and a reduced IC_50_ value.	[167]

**Table 6:** A list of patents for chitosan-based NPs for cancer treatment.

Patent Number	Title of Patent	Description
CN103520730A	Chitosan oligosaccharide nanoparticles capable of loading hydrophobic anti-tumor medicine	It provided improved targeting properties, slow drug release, and low toxicity.
CN112675150B	Preparation method of tumor-targeted drug-loaded nanoparticles based on stibene	It causes tumor targeting, slow release of the drug, and increased stability of the loaded drug. In addition, it can realize fluorescence imaging, photoacoustic imaging, and photo-thermal/chemotherapy synergistic treatment.
US20110190399A1	Curcumin nanoparticles and methods of producing the same	It provided 10 times greater bioavailability of curcumin than a simple solution.
CN111214460A	Folic acid-chitosan-nano-selenium tumor-targeted drug delivery system and preparation method thereof	It has the advantages of reduced size, uniform particle size distribution, easy realization of the method, stable property, and strong repeatability
KR20120083701A	Nanoparticles are formed by encapsulating an anticancer drug into a glycolchitosan-cholanic acid complex, and a process for the preparation thereof	It provided greater solubility in water, high efficiency, low toxicity, and sterilizing filtration
US788372382	Water-soluble chitosan nanoparticle for delivering an anticancer agent and preparing a method thereof	It provided efficient delivery of paclitaxel by incorporating hydrophilic and hydrophobic functional groups into the highly reactive amine group of the water-soluble chitosan
WO2014130866A3	Targeted buccal delivery comprising cisplatin-loaded chitosan nanoparticles	It provided a diagnostic, therapeutic, or delivery system for local and systemic administration through the oral epithelial cells
CN103169665A	Oxaliplatin chitosan nanoparticle and application thereof	It has a reduced grain size, high encapsulation efficiency, a low material price, and is simple

## Data Availability

Not applicable.

## References

[1] Grover M., Behl T., Virmani T., Sanduja M., Makeen H.A., Albratty M., Alhazmi H.A., Meraya A.M., Bungau S.G. (2022). Exploration of Cytotoxic Potential of Longifolene/Junipene Isolated from Chrysopogon Zizanioides. Molecules.

[2] Joyce Nirmala M., Kizhuveetil U., Johnson A., Balaji G., Nagarajan R., Muthuvijayan V. (2023). Cancer Nanomedicine: A Review of Nano-Therapeutics and Challenges Ahead. RSC Adv..

[3] Grover M., Behl T., Virmani T. (2021). Phytochemical Screening, Antioxidant Assay and Cytotoxic Profile for Different Extracts of Chrysopogon Zizanioides Roots. Chem. Biodivers.

[4] Shah S.C., Kayamba V., Peek R.M., Heimburger D. (2019). Cancer Control in Low- and Middle-Income Countries: Is It Time to Consider Screening?. J. Glob. Oncol..

[5] Brinks J., Fowler A., Franklin B.A., Dulai J. (2017). Lifestyle Modification in Secondary Prevention: Beyond Pharmacotherapy. Am. J. Lifestyle Med..

[6] Gao Q., Feng J., Liu W., Wen C., Wu Y., Liao Q., Zou L., Sui X., Xie T., Zhang J. (2022). Opportunities and Challenges for Co-Delivery Nanomedicines Based on Combination of Phytochemicals with Chemotherapeutic Drugs in Cancer Treatment. Adv. Drug Deliv. Rev..

[7] Anand U., Dey A., Chandel A.K.S., Sanyal R., Mishra A., Pandey D.K., De Falco V., Upadhyay A., Kandimalla R., Chaudhary A. (2022). Cancer Chemotherapy and beyond: Current Status, Drug Candidates, Associated Risks and Progress in Targeted Therapeutics. Genes Dis..

[8] Kumar G., Virmani T., Sharma A., Pathak K. (2023). Codelivery of Phytochemicals with Conventional Anticancer Drugs in Form of Nanocarriers. Pharmaceutics.

[9] Shakil M.S., Mahmud K.M., Sayem M., Niloy M.S., Halder S.K., Hossen M.S., Uddin M.F., Hasan M.A. (2021). Using Chitosan or Chitosan Derivatives in Cancer Therapy. Polysaccharides.

[10] Kumar G., Khar R.K., Virmani T., Jogpal V., Virmani R. (2018). Comparative Evaluation of Fast Dissolving Tablet of Atorvastatin Calcium Using Natural and Synthetic Super Disintegrating Agents. Res. J. Pharm. Technol..

[11] Kumar G., Virmani T., Pathak K., Alhalmi A. (2022). A Revolutionary Blueprint for Mitigation of Hypertension via Nanoemulsion. BioMed Res. Int..

[12] Virmani T., Kumar G., Pathak K. Non-Aqueous Nanoemulsions: An Innovative Lipid-Based Drug Carrier. https://www.igi-global.com/chapter/non-aqueous-nanoemulsions/www.igi-global.com/chapter/non-aqueous-nanoemulsions/300404.

[13] Zahin N., Anwar R., Tewari D., Kabir M.T., Sajid A., Mathew B., Uddin M.S., Aleya L., Abdel-Daim M.M. (2020). Nanoparticles and Its Biomedical Applications in Health and Diseases: Special Focus on Drug Delivery. Environ. Sci. Pollut. Res..

[14] Sahu T., Ratre Y.K., Chauhan S., Bhaskar L.V.K.S., Nair M.P., Verma H.K. (2021). Nanotechnology Based Drug Delivery System: Current Strategies and Emerging Therapeutic Potential for Medical Science. J. Drug Deliv. Sci. Technol..

[15] Patra J.K., Das G., Fraceto L.F., Campos E.V.R., Rodriguez-Torres M.d.P., Acosta-Torres L.S., Diaz-Torres L.A., Grillo R., Swamy M.K., Sharma S. (2018). Nano Based Drug Delivery Systems: Recent Developments and Future Prospects. J Nanobiotechnol..

[16] Naqvi S., Panghal A., Flora S.J.S. (2020). Nanotechnology: A Promising Approach for Delivery of Neuroprotective Drugs. Front. Neurosci..

[17] Virmani T., Kumar G., Virmani R., Sharma A., Pathak K. (2022). Nanocarrier-Based Approaches to Combat Chronic Obstructive Pulmonary Disease. Nanomedicine.

[18] Kumar G., Virmani T., Pathak K., Kamaly O.A., Saleh A. (2022). Central Composite Design Implemented Azilsartan Medoxomil Loaded Nanoemulsion to Improve Its Aqueous Solubility and Intestinal Permeability: In Vitro and Ex Vivo Evaluation. Pharmaceuticals.

[19] Perumal S. (2022). Polymer Nanoparticles: Synthesis and Applications. Polymers.

[20] Zielińska A., Carreiró F., Oliveira A.M., Neves A., Pires B., Venkatesh D.N., Durazzo A., Lucarini M., Eder P., Silva A.M. (2020). Polymeric Nanoparticles: Production, Characterization, Toxicology and Ecotoxicology. Molecules.

[21] Colone M., Calcabrini A., Stringaro A. (2020). Drug Delivery Systems of Natural Products in Oncology. Molecules.

[22] Chang D., Ma Y., Xu X., Xie J., Ju S. (2021). Stimuli-Responsive Polymeric Nanoplatforms for Cancer Therapy. Front. Bioeng. Biotechnol..

[23] Virmani T., Kumar G., Virmani R., Sharma A., Pathak K. (2023). Xanthan Gum-Based Drug Delivery Systems for Respiratory Diseases. Natural Polymeric Materials Based Drug Delivery Systems in Lung Diseases.

[24] Polat M., Polat H. (2019). Recent Advances in Chitosan-Based Systems for Delivery of Anticancer Drugs. Functional Chitosan: Drug Delivery and Biomedical Applications.

[25] Herdiana Y., Wathoni N., Shamsuddin S., Joni I.M., Muchtaridi M. (2021). Chitosan-Based Nanoparticles of Targeted Drug Delivery System in Breast Cancer Treatment. Polymers.

[26] Almutairi F.M., Abd-Rabou A.A., Mohamed M.S. (2019). Raloxifene-Encapsulated Hyaluronic Acid-Decorated Chitosan Nanoparticles Selectively Induce Apoptosis in Lung Cancer Cells. Bioorganic Med. Chem..

[27] Ullah S., Azad A.K., Nawaz A., Shah K.U., Iqbal M., Albadrani G.M., Al-Joufi F.A., Sayed A.A., Abdel-Daim M.M. (2022). 5-Fluorouracil-Loaded Folic-Acid-Fabricated Chitosan Nanoparticles for Site-Targeted Drug Delivery Cargo. Polymers.

[28] Bosch F., Rosich L. (2008). The Contributions of Paul Ehrlich to Pharmacology: A Tribute on the Occasion of the Centenary of His Nobel Prize. Pharmacology.

[29] Arruebo M., Vilaboa N., Sáez-Gutierrez B., Lambea J., Tres A., Valladares M., González-Fernández Á. (2011). Assessment of the Evolution of Cancer Treatment Therapies. Cancers.

[30] Dehelean C.A., Marcovici I., Soica C., Mioc M., Coricovac D., Iurciuc S., Cretu O.M., Pinzaru I. (2021). Plant-Derived Anticancer Compounds as New Perspectives in Drug Discovery and Alternative Therapy. Molecules.

[31] Gupta V., Virmani D.T., Singh D.V. (2022). A Comparative Diagnostic Account of the Roots of Boerhavia Diffusa Linn. From Four Different Geographical Regions in India. J. Community Pharm. Pract. (JCPP) 2799-1199.

[32] Talib W.H., Awajan D., Hamed R.A., Azzam A.O., Mahmod A.I., AL-Yasari I.H. (2022). Combination Anticancer Therapies Using Selected Phytochemicals. Molecules.

[33] Siddiqui A.J., Jahan S., Singh R., Saxena J., Ashraf S.A., Khan A., Choudhary R.K., Balakrishnan S., Badraoui R., Bardakci F. (2022). Plants in Anticancer Drug Discovery: From Molecular Mechanism to Chemoprevention. BioMed Res. Int..

[34] Alqosaibi A.I. (2022). Nanocarriers for Anticancer Drugs: Challenges and Perspectives. Saudi J. Biol. Sci..

[35] Gyanani V., Haley J.C., Goswami R. (2021). Challenges of Current Anticancer Treatment Approaches with Focus on Liposomal Drug Delivery Systems. Pharmaceuticals.

[36] Mansoori B., Mohammadi A., Davudian S., Shirjang S., Baradaran B. (2017). The Different Mechanisms of Cancer Drug Resistance: A Brief Review. Adv. Pharm. Bull..

[37] ALTUN İ., SONKAYA A. (2018). The Most Common Side Effects Experienced by Patients Were Receiving First Cycle of Chemotherapy. Iran J. Public Health.

[38] Edis Z., Wang J., Waqas M.K., Ijaz M., Ijaz M. (2021). Nanocarriers-Mediated Drug Delivery Systems for Anticancer Agents: An Overview and Perspectives. Int. J. Nanomed..

[39] Manzari M.T., Shamay Y., Kiguchi H., Rosen N., Scaltriti M., Heller D.A. (2021). Targeted Drug Delivery Strategies for Precision Medicines. Nat. Rev. Mater..

[40] Senapati S., Mahanta A.K., Kumar S., Maiti P. (2018). Controlled Drug Delivery Vehicles for Cancer Treatment and Their Performance. Signal Transduct. Target Ther..

[41] Xiao X., Teng F., Shi C., Chen J., Wu S., Wang B., Meng X., Essiet Imeh A., Li W. (2022). Polymeric Nanoparticles—Promising Carriers for Cancer Therapy. Front. Bioeng. Biotechnol..

[42] Herdiana Y., Wathoni N., Gozali D., Shamsuddin S., Muchtaridi M. (2023). Chitosan-Based Nano-Smart Drug Delivery System in Breast Cancer Therapy. Pharmaceutics.

[43] Karam M., Fahs D., Maatouk B., Safi B., Jaffa A.A., Mhanna R. (2022). Polymeric Nanoparticles in the Diagnosis and Treatment of Myocardial Infarction: Challenges and Future Prospects. Mater. Today Bio.

[44] Díez-Pascual A.M. (2022). Surface Engineering of Nanomaterials with Polymers, Biomolecules, and Small Ligands for Nanomedicine. Materials.

[45] Komsthöft T., Bovone G., Bernhard S., Tibbitt M.W. (2022). Polymer Functionalization of Inorganic Nanoparticles for Biomedical Applications. Curr. Opin. Chem. Eng..

[46] Aranaz I., Alcántara A.R., Civera M.C., Arias C., Elorza B., Heras Caballero A., Acosta N. (2021). Chitosan: An Overview of Its Properties and Applications. Polymers.

[47] Lim C., Hwang D.S., Lee D.W. (2021). Intermolecular Interactions of Chitosan: Degree of Acetylation and Molecular Weight. Carbohydr. Polym..

[48] Elgadir M.A., Uddin M.S., Ferdosh S., Adam A., Chowdhury A.J.K., Sarker M.Z.I. (2015). Impact of Chitosan Composites and Chitosan Nanoparticle Composites on Various Drug Delivery Systems: A Review. J. Food Drug Anal..

[49] Chen Y.-Z., Huang Y.-K., Chen Y., Ye Y.-J., Lou K.-Y., Gao F. (2015). Novel Nanoparticles Composed of Chitosan and β-Cyclodextrin Derivatives as Potential Insoluble Drug Carrier. Chin. Chem. Lett..

[50] Mikušová V., Mikuš P. (2021). Advances in Chitosan-Based Nanoparticles for Drug Delivery. Int. J. Mol. Sci..

[51] Jafernik K., Ładniak A., Blicharska E., Czarnek K., Ekiert H., Wiącek A.E., Szopa A. (2023). Chitosan-Based Nanoparticles as Effective Drug Delivery Systems—A Review. Molecules.

[52] Baharlouei P., Rahman A. (2022). Chitin and Chitosan: Prospective Biomedical Applications in Drug Delivery, Cancer Treatment, and Wound Healing. Mar. Drugs.

[53] Ways T.M., Lau W., Khutoryanskiy V. (2018). Chitosan and Its Derivatives for Application in Mucoadhesive Drug Delivery Systems. Polymers.

[54] Kumar S., Deepak V., Kumari M., Dutta P. (2015). Antibacterial Activity of Diisocyanate-Modified Chitosan for Biomedical Applications. Int. J. Biol. Macromol..

[55] Xing L., Fan Y.-T., Zhou T.-J., Gong J.-H., Cui L.-H., Cho K.-H., Choi Y.-J., Jiang H.-L., Cho C.-S. (2018). Chemical Modification of Chitosan for Efficient Vaccine Delivery. Molecules.

[56] Pellis A., Guebitz G.M., Nyanhongo G.S. (2022). Chitosan: Sources, Processing and Modification Techniques. Gels.

[57] Chen Q., Qi Y., Jiang Y., Quan W., Luo H., Wu K., Li S., Ouyang Q. (2022). Progress in Research of Chitosan Chemical Modification Technologies and Their Applications. Mar. Drugs.

[58] Hamedi H., Moradi S., Hudson S.M., Tonelli A.E. (2018). Chitosan Based Hydrogels and Their Applications for Drug Delivery in Wound Dressings: A Review. Carbohydr. Polym..

[59] Ali A., Ahmed S. (2018). A Review on Chitosan and Its Nanocomposites in Drug Delivery. Int. J. Biol. Macromol..

[60] Safdar R., Omar A.A., Arunagiri A., Regupathi I., Thanabalan M. (2019). Potential of Chitosan and Its Derivatives for Controlled Drug Release Applications—A Review. J. Drug Deliv. Sci. Technol..

[61] Zamboulis A., Nanaki S., Michailidou G., Koumentakou I., Lazaridou M., Ainali N.M., Xanthopoulou E., Bikiaris D.N. (2020). Chitosan and Its Derivatives for Ocular Delivery Formulations: Recent Advances and Developments. Polymers.

[62] Arya G., Gupta N., Nimesh S., Venkatesan J., Kim S.-K., Anil S.P.d.R. (2022). 8—Chitosan Nanoparticles for Therapeutic Delivery of Anticancer Drugs. Polysaccharide Nanoparticles.

[63] Kurczewska J. (2023). Chitosan-Based Nanoparticles with Optimized Parameters for Targeted Delivery of a Specific Anticancer Drug—A Comprehensive Review. Pharmaceutics.

[64] Botelho Da Silva S., Krolicka M., Van Den Broek L.A.M., Frissen A.E., Boeriu C.G. (2018). Water-Soluble Chitosan Derivatives and PH-Responsive Hydrogels by Selective C-6 Oxidation Mediated by TEMPO-Laccase Redox System. Carbohydr. Polym..

[65] Babu A., Ramesh R. (2017). Multifaceted Applications of Chitosan in Cancer Drug Delivery and Therapy. Mar. Drugs.

[66] Jaiswal S., Dutta P.K., Kumar S., Chawla R. (2021). Chitosan Modified by Organo-Functionalities as an Efficient Nanoplatform for Anti-Cancer Drug Delivery Process. J. Drug Deliv. Sci. Technol..

[67] Carroll E.C., Jin L., Mori A., Muñoz-Wolf N., Oleszycka E., Moran H.B.T., Mansouri S., McEntee C.P., Lambe E., Agger E.M. (2016). The Vaccine Adjuvant Chitosan Promotes Cellular Immunity via DNA Sensor CGAS-STING-Dependent Induction of Type I Interferons. Immunity.

[68] Li W., Zhu X., Zhou X., Wang X., Zhai W., Li B., Du J., Li G., Sui X., Wu Y. (2021). An Orally Available PD-1/PD-L1 Blocking Peptide OPBP-1-Loaded Trimethyl Chitosan Hydrogel for Cancer Immunotherapy. J. Control. Release.

[69] Esmaily M., Masjedi A., Hallaj S., Nabi Afjadi M., Malakotikhah F., Ghani S., Ahmadi A., Sojoodi M., Hassannia H., Atyabi F. (2020). Blockade of CTLA-4 Increases Anti-Tumor Response Inducing Potential of Dendritic Cell Vaccine. J Control. Release.

[70] Narmani A., Jafari S.M. (2021). Chitosan-Based Nanodelivery Systems for Cancer Therapy: Recent Advances. Carbohydr. Polym..

[71] Abruzzo A., Zuccheri G., Belluti F., Provenzano S., Verardi L., Bigucci F., Cerchiara T., Luppi B., Calonghi N. (2016). Chitosan Nanoparticles for Lipophilic Anticancer Drug Delivery: Development, Characterization and in Vitro Studies on HT29 Cancer Cells. Colloids Surf. B Biointerfaces.

[72] Chen G., Svirskis D., Lu W., Ying M., Huang Y., Wen J. (2018). N-Trimethyl Chitosan Nanoparticles and CSKSSDYQC Peptide: N-Trimethyl Chitosan Conjugates Enhance the Oral Bioavailability of Gemcitabine to Treat Breast Cancer. J. Control. Release.

[73] Vllasaliu D., Exposito-Harris R., Heras A., Casettari L., Garnett M., Illum L., Stolnik S. (2010). Tight Junction Modulation by Chitosan Nanoparticles: Comparison with Chitosan Solution. Int. J. Pharm..

[74] Alhodieb F.S., Barkat M.A., Barkat H.A., Hadi H.A., Khan M.I., Ashfaq F., Rahman M.A., Hassan M.Z., Alanezi A.A. (2022). Chitosan-Modified Nanocarriers as Carriers for Anticancer Drug Delivery: Promises and Hurdles. Int. J. Biol. Macromol..

[75] Jha R., Mayanovic R.A. (2023). A Review of the Preparation, Characterization, and Applications of Chitosan Nanoparticles in Nanomedicine. Nanomaterials.

[76] Hossen S., Hossain M.K., Basher M.K., Mia M.N.H., Rahman M.T., Uddin M.J. (2019). Smart Nanocarrier-Based Drug Delivery Systems for Cancer Therapy and Toxicity Studies: A Review. J. Adv. Res..

[77] Abd Ellah N.H., Abouelmagd S.A. (2017). Surface Functionalization of Polymeric Nanoparticles for Tumor Drug Delivery: Approaches and Challenges. Expert Opin. Drug Deliv..

[78] Batlle R., Andrés E., Gonzalez L., Llonch E., Igea A., Gutierrez-Prat N., Berenguer-Llergo A., Nebreda A.R. (2019). Regulation of Tumor Angiogenesis and Mesenchymal-Endothelial Transition by P38α through TGF-β and JNK Signaling. Nat. Commun..

[79] Yermak I.M., Davydova V.N., Volod’ko A.V. (2022). Mucoadhesive Marine Polysaccharides. Mar. Drugs.

[80] Bae Y.H., Park K. (2020). Advanced Drug Delivery 2020 and beyond: Perspectives on the Future. Adv. Drug Deliv. Rev..

[81] Fang J., Islam W., Maeda H. (2020). Exploiting the Dynamics of the EPR Effect and Strategies to Improve the Therapeutic Effects of Nanomedicines by Using EPR Effect Enhancers. Adv. Drug Deliv. Rev..

[82] Mohammed M.A., Syeda J.T.M., Wasan K.M., Wasan E.K. (2017). An Overview of Chitosan Nanoparticles and Its Application in Non-Parenteral Drug Delivery. Pharmaceutics.

[83] Zhao X., Si J., Huang D., Li K., Xin Y., Sui M. (2020). Application of Star Poly(Ethylene Glycol) Derivatives in Drug Delivery and Controlled Release. J. Control. Release.

[84] Zhou Z., Liu Y., Jiang X., Zheng C., Luo W., Xiang X., Qi X., Shen J. (2023). Metformin Modified Chitosan as a Multi-Functional Adjuvant to Enhance Cisplatin-Based Tumor Chemotherapy Efficacy. Int. J. Biol. Macromol..

[85] Zhou Z., Zheng C., Liu Y., Luo W., Deng H., Shen J. (2022). Chitosan Biguanide Induced Mitochondrial Inhibition to Amplify the Efficacy of Oxygen-Sensitive Tumor Therapies. Carbohydr. Polym..

[86] Chen J., Zhou Z., Zheng C., Liu Y., Hao R., Ji X., Xi Q., Shen J., Li Z. (2022). Chitosan Oligosaccharide Regulates AMPK and STAT1 Pathways Synergistically to Mediate PD-L1 Expression for Cancer Chemoimmunotherapy. Carbohydr. Polym..

[87] Cai X., Feng J., Chen F., Guo C., Sun L., Li L. (2020). Synergistic Effect of Glycated Chitosan and Photofrin Photodynamic Therapy on Different Breast Tumor Model. Photodiagnosis Photodyn. Ther..

[88] Ding J., Guo Y. (2022). Recent Advances in Chitosan and Its Derivatives in Cancer Treatment. Front. Pharm..

[89] Li X., Dong W., Nalin A.P., Wang Y., Pan P., Xu B., Zhang Y., Tun S., Zhang J., Wang L.-S. (2018). The Natural Product Chitosan Enhances the Anti-Tumor Activity of Natural Killer Cells by Activating Dendritic Cells. Oncoimmunology.

[90] Mortezaee K., Narmani A., Salehi M., Bagheri H., Farhood B., Haghi-Aminjan H., Najafi M. (2021). Synergic Effects of Nanoparticles-Mediated Hyperthermia in Radiotherapy/Chemotherapy of Cancer. Life Sci..

[91] Fernández M., Javaid F., Chudasama V. (2018). Advances in Targeting the Folate Receptor in the Treatment/Imaging of Cancers. Chem. Sci..

[92] Narmani A., Rezvani M., Farhood B., Darkhor P., Mohammadnejad J., Amini B., Refahi S., Abdi Goushbolagh N. (2019). Folic Acid Functionalized Nanoparticles as Pharmaceutical Carriers in Drug Delivery Systems. Drug Dev. Res..

[93] Dunn M.R., Jimenez R.M., Chaput J.C. (2017). Analysis of Aptamer Discovery and Technology. Nat. Rev. Chem..

[94] Tang Y., Wu S., Lin J., Cheng L., Zhou J., Xie J., Huang K., Wang X., Yu Y., Chen Z. (2018). Nanoparticles Targeted against Cryptococcal Pneumonia by Interactions between Chitosan and Its Peptide Ligand. Nano Lett..

[95] Kamalabadi-Farahani M., Vasei M., Ahmadbeigi N., Ebrahimi-Barough S., Soleimani M., Roozafzoon R. (2018). Anti-Tumour Effects of TRAIL-Expressing Human Placental Derived Mesenchymal Stem Cells with Curcumin-Loaded Chitosan Nanoparticles in a Mice Model of Triple Negative Breast Cancer. Artif. Cells Nanomed. Biotechnol..

[96] Kavaz D., Kirac F., Kirac M., Vaseashta A. (2017). Low Releasing Mitomycin C Molecule Encapsulated with Chitosan Nanoparticles for Intravesical Installation. J. Biomater. Nanobiotechnol..

[97] Komenek S., Luesakul U., Ekgasit S., Vilaivan T., Praphairaksit N., Puthong S., Muangsin N. (2017). Nanogold-Gallate Chitosan-Targeted Pulmonary Delivery for Treatment of Lung Cancer. AAPS PharmSciTech..

[98] Alkhader E., Billa N., Roberts C.J. (2017). Mucoadhesive Chitosan-Pectinate Nanoparticles for the Delivery of Curcumin to the Colon. AAPS PharmSciTech..

[99] Gupta U., Sharma S., Khan I., Gothwal A., Sharma A.K., Singh Y., Chourasia M.K., Kumar V. (2017). Enhanced Apoptotic and Anticancer Potential of Paclitaxel Loaded Biodegradable Nanoparticles Based on Chitosan. Int. J. Biol. Macromol..

[100] Manivasagan P., Bharathiraja S., Bui N.Q., Lim I.G., Oh J. (2016). Paclitaxel-Loaded Chitosan Oligosaccharide-Stabilized Gold Nanoparticles as Novel Agents for Drug Delivery and Photoacoustic Imaging of Cancer Cells. Int. J. Pharm..

[101] Motawi T.K., El-Maraghy S.A., ElMeshad A.N., Nady O.M., Hammam O.A. (2017). Cromolyn Chitosan Nanoparticles as a Novel Protective Approach for Colorectal Cancer. Chem. Biol. Interact..

[102] Zare M., Mohammadi Samani S., Sobhani Z. (2018). Enhanced Intestinal Permeation of Doxorubicin Using Chitosan Nanoparticles. Adv. Pharm. Bull..

[103] Helmi O., Elshishiny F., Mamdouh W. (2021). Targeted Doxorubicin Delivery and Release within Breast Cancer Environment Using PEGylated Chitosan Nanoparticles Labeled with Monoclonal Antibodies. Int. J. Biol. Macromol..

[104] Farrag N.S., Shetta A., Mamdouh W. (2021). Green Tea Essential Oil Encapsulated Chitosan Nanoparticles-Based Radiopharmaceutical as a New Trend for Solid Tumor Theranosis. Int. J. Biol. Macromol..

[105] Yadav A.S., Radharani N.N.V., Gorain M., Bulbule A., Shetti D., Roy G., Baby T., Kundu G.C. (2020). RGD Functionalized Chitosan Nanoparticle Mediated Targeted Delivery of Raloxifene Selectively Suppresses Angiogenesis and Tumor Growth in Breast Cancer. Nanoscale.

[106] Murthy A., Ravi P.R., Kathuria H., Vats R. (2020). Self-Assembled Lecithin-Chitosan Nanoparticles Improve the Oral Bioavailability and Alter the Pharmacokinetics of Raloxifene. Int. J. Pharm..

[107] Zhang W., Xu W., Lan Y., He X., Liu K., Liang Y. (2019). Antitumor Effect of Hyaluronic-Acid-Modified Chitosan Nanoparticles Loaded with SiRNA for Targeted Therapy for Non-Small Cell Lung Cancer. Int. J. Nanomed..

[108] Jain A., Thakur K., Kush P., Jain U.K. (2014). Docetaxel Loaded Chitosan Nanoparticles: Formulation, Characterization and Cytotoxicity Studies. Int. J. Biol. Macromol..

[109] Mirzaie Z.H., Irani S., Mirfakhraie R., Atyabi S.M., Dinarvand M., Dinarvand R., Varshochian R., Atyabi F. (2016). Docetaxel-Chitosan Nanoparticles for Breast Cancer Treatment: Cell Viability and Gene Expression Study. Chem. Biol. Drug Des..

[110] Hua S., Yu J., Shang J., Zhang H., Du J., Zhang Y., Chen F., Zhou Y., Liu F. (2016). Effective Tumor-Targeted Delivery of Etoposide Using Chitosan Nanoparticles Conjugated with Folic Acid and Sulfobetaine Methacrylate. RSC Adv..

[111] Barbosa A.I., Costa Lima S.A., Reis S. (2019). Development of Methotrexate Loaded Fucoidan/Chitosan Nanoparticles with Anti-Inflammatory Potential and Enhanced Skin Permeation. Int. J. Biol. Macromol..

[112] Ekinci M., Ilem-Ozdemir D., Gundogdu E., Asikoglu M. (2015). Methotrexate Loaded Chitosan Nanoparticles: Preparation, Radiolabeling and in Vitro Evaluation for Breast Cancer Diagnosis. J. Drug Deliv. Sci. Technol..

[113] Sultan M.H., Moni S.S., Madkhali O.A., Bakkari M.A., Alshahrani S., Alqahtani S.S., Alhakamy N.A., Mohan S., Ghazwani M., Bukhary H.A. (2022). Characterization of Cisplatin-Loaded Chitosan Nanoparticles and Rituximab-Linked Surfaces as Target-Specific Injectable Nano-Formulations for Combating Cancer. Sci. Rep..

[114] Sun L., Chen Y., Zhou Y., Guo D., Fan Y., Guo F., Zheng Y., Chen W. (2017). Preparation of 5-Fluorouracil-Loaded Chitosan Nanoparticles and Study of the Sustained Release in Vitro and in Vivo. Asian J. Pharm. Sci..

[115] Patel G., Yadav B.K.N. (2020). Study of 5-Fluorouracil Loaded Chitosan Nanoparticles for Treatment of Skin Cancer. Recent Pat. Nanotechnol..

[116] Shayegh A., Khalatbari F., Zonoubi N., Zarazvand F., Monavvari F., Hejazinia H., Ebrahimi S.E.S., Hamedani M.P., Ali V., Hadadian S. (2021). Chlorambucil-Chitosan Nano-Conjugate: An Efficient Agent Against Breast Cancer Targeted Therapy. Curr. Drug Deliv..

[117] Kaur H., Mishra N., Khurana B., Kaur S., Arora D. (2021). DoE Based Optimization and Development of Spray-Dried Chitosan-Coated Alginate Microparticles Loaded with Cisplatin for the Treatment of Cervical Cancer. Curr. Mol. Pharm..

[118] Hemmati K., Ahmadi Nasab N., Hesaraki S., Nezafati N. (2021). In Vitro Evaluation of Curcumin-Loaded Chitosan-Coated Hydroxyapatite Nanocarriers as a Potential System for Effective Treatment of Cancer. J. Biomater. Sci. Polym. Ed..

[119] Nokhodi F., Nekoei M., Goodarzi M.T. (2022). Hyaluronic Acid-Coated Chitosan Nanoparticles as Targeted-Carrier of Tamoxifen against MCF7 and TMX-Resistant MCF7 Cells. J. Mater. Sci. Mater. Med..

[120] Cannavà C., De Gaetano F., Stancanelli R., Venuti V., Paladini G., Caridi F., Ghica C., Crupi V., Majolino D., Ferlazzo G. (2022). Chitosan-Hyaluronan Nanoparticles for Vinblastine Sulfate Delivery: Characterization and Internalization Studies on K-562 Cells. Pharmaceutics.

[121] Gooneh-Farahani S., Naghib S.M., Naimi-Jamal M.R., Seyfoori A. (2021). A PH-Sensitive Nanocarrier Based on BSA-Stabilized Graphene-Chitosan Nanocomposite for Sustained and Prolonged Release of Anticancer Agents. Sci. Rep..

[122] Yi G., Ling J., Jiang Y., Lu Y., Yang L.-Y., Ouyang X. (2022). Fabrication, Characterization, and in Vitro Evaluation of Doxorubicin-Coupled Chitosan Oligosaccharide Nanoparticles. J. Mol. Struct..

[123] Scheeren L.E., Nogueira D.R., Macedo L.B., Vinardell M.P., Mitjans M., Infante M.R., Rolim C.M.B. (2016). PEGylated and Poloxamer-Modified Chitosan Nanoparticles Incorporating a Lysine-Based Surfactant for PH-Triggered Doxorubicin Release. Colloids Surf. B Biointerfaces.

[124] Varukattu N.B., Vivek R., Rejeeth C., Thangam R., Ponraj T., Sharma A., Kannan S. (2020). Nanostructured PH-Responsive Biocompatible Chitosan Coated Copper Oxide Nanoparticles: A Polymeric Smart Intracellular Delivery System for Doxorubicin in Breast Cancer Cells. Arab. J. Chem..

[125] Luckanagul J.A., Pitakchatwong C., Ratnatilaka Na Bhuket P., Muangnoi C., Rojsitthisak P., Chirachanchai S., Wang Q., Rojsitthisak P. (2018). Chitosan-Based Polymer Hybrids for Thermo-Responsive Nanogel Delivery of Curcumin. Carbohydr. Polym..

[126] Hu Y., Du Y., Liu N., Liu X., Meng T., Cheng B., He J., You J., Yuan H., Hu F. (2015). Selective Redox-Responsive Drug Release in Tumor Cells Mediated by Chitosan Based Glycolipid-like Nanocarrier. J. Control. Release.

[127] Xu W., Wang H., Dong L., Zhang P., Mu Y., Cui X., Zhou J., Huo M., Yin T. (2019). Hyaluronic Acid-Decorated Redox-Sensitive Chitosan Micelles for Tumor-Specific Intracellular Delivery of Gambogic Acid. Int. J. Nanomed..

[128] Qi M., Li G., Yu N., Meng Y., Liu X. (2014). Synthesis of Thermo-Sensitive Polyelectrolyte Complex Nanoparticles from CS-g-PNIPAM and SA-g-PNIPAM for Controlled Drug Release. Macromol. Res..

[129] Yan L., Crayton S.H., Thawani J.P., Amirshaghaghi A., Tsourkas A., Cheng Z. (2015). A PH-Responsive Drug-Delivery Platform Based on Glycol Chitosan–Coated Liposomes. Small.

[130] Mansouri M., Nazarpak M.H., Solouk A., Akbari S., Hasani-Sadrabadi M.M. (2017). Magnetic Responsive of Paclitaxel Delivery System Based on SPION and Palmitoyl Chitosan. J. Magn. Magn. Mater..

[131] Zhong S., Zhang H., Liu Y., Wang G., Shi C., Li Z., Feng Y., Cui X. (2017). Folic Acid Functionalized Reduction-Responsive Magnetic Chitosan Nanocapsules for Targeted Delivery and Triggered Release of Drugs. Carbohydr. Polym..

[132] Meng L., Huang W., Wang D., Huang X., Zhu X., Yan D. (2013). Chitosan-Based Nanocarriers with PH and Light Dual Response for Anticancer Drug Delivery. Biomacromolecules.

[133] El-Leithy E.S., Hassan S.A., Abdel-Rashid R.S. (2019). Tamoxifen Citrate/Coenzyme Q10 as Smart Nanocarriers Bitherapy for Breast Cancer: Cytotoxicity, Genotoxicity, and Antioxidant Activity. J. Drug Deliv. Sci. Technol..

[134] Li P., Wang Y., Peng Z., She F., Kong L. (2011). Development of Chitosan Nanoparticles as Drug Delivery Systems for 5-Fluorouracil and Leucovorin Blends. Carbohydr. Polym..

[135] Jia M., Li Y., Yang X., Huang Y., Wu H., Huang Y., Lin J., Li Y., Hou Z., Zhang Q. (2014). Development of Both Methotrexate and Mitomycin C Loaded PEGylated Chitosan Nanoparticles for Targeted Drug Codelivery and Synergistic Anticancer Effect. ACS Appl. Mater. Interfaces.

[136] Khan M.M., Madni A., Tahir N., Parveen F., Khan S., Jan N., Ali A., Abdurrahim M., Farooq U., Khan M.I. (2020). Co-Delivery of Curcumin and Cisplatin to Enhance Cytotoxicity of Cisplatin Using Lipid-Chitosan Hybrid Nanoparticles. Int. J. Nanomed..

[137] Deng X., Cao M., Zhang J., Hu K., Yin Z., Zhou Z., Xiao X., Yang Y., Sheng W., Wu Y. (2014). Hyaluronic Acid-Chitosan Nanoparticles for Co-Delivery of MiR-34a and Doxorubicin in Therapy against Triple Negative Breast Cancer. Biomaterials.

[138] Chen G., Zhao Y., Xu Y., Zhu C., Liu T., Wang K. (2020). Chitosan Nanoparticles for Oral Photothermally Enhanced Photodynamic Therapy of Colon Cancer. Int. J. Pharm..

[139] Anitha A., Deepa N., Chennazhi K.P., Lakshmanan V.-K., Jayakumar R. (2014). Combinatorial Anticancer Effects of Curcumin and 5-Fluorouracil Loaded Thiolated Chitosan Nanoparticles towards Colon Cancer Treatment. Biochim. Et Biophys. Acta (BBA) Gen. Subj..

[140] Sadreddini S., Safaralizadeh R., Baradaran B., Aghebati-Maleki L., Hosseinpour-Feizi M.A., Shanehbandi D., Jadidi-Niaragh F., Sadreddini S., Kafil H.S., Younesi V. (2017). Chitosan Nanoparticles as a Dual Drug/SiRNA Delivery System for Treatment of Colorectal Cancer. Immunol. Lett..

[141] Mohammadi Z., Samadi F.Y., Rahmani S., Mohammadi Z. (2020). Chitosan-Raloxifene Nanoparticle Containing Doxorubicin as a New Double-Effect Targeting Vehicle for Breast Cancer Therapy. Daru.

[142] Zhu X., Yu Z., Feng L., Deng L., Fang Z., Liu Z., Li Y., Wu X., Qin L., Guo R. (2021). Chitosan-Based Nanoparticle Co-Delivery of Docetaxel and Curcumin Ameliorates Anti-Tumor Chemoimmunotherapy in Lung Cancer. Carbohydr. Polym..

[143] Fahmy S.A., Ramzy A., Mandour A.A., Nasr S., Abdelnaser A., Bakowsky U., Azzazy H.M.E.-S. (2022). PEGylated Chitosan Nanoparticles Encapsulating Ascorbic Acid and Oxaliplatin Exhibit Dramatic Apoptotic Effects against Breast Cancer Cells. Pharmaceutics.

[144] Xin L., Fan J.-C., Le Y.-G., Zeng F., Cheng H., Hu X., Cao J.-Q. (2016). Construction of METHFR ShRNA/5-Fluorouracil Co-Loaded Folate-Targeted Chitosan Polymeric Nanoparticles and Its Anti-Carcinoma Effect on Gastric Cells Growth. J. Nanopart. Res..

[145] Sebaaly C., Trifan A., Sieniawska E., Greige-Gerges H. (2021). Chitosan-Coating Effect on the Characteristics of Liposomes: A Focus on Bioactive Compounds and Essential Oils: A Review. Processes.

[146] Priya S., Desai V.M., Singhvi G. (2023). Surface Modification of Lipid-Based Nanocarriers: A Potential Approach to Enhance Targeted Drug Delivery. ACS Omega.

[147] Ganesan P., Ramalingam P., Karthivashan G., Ko Y.T., Choi D.-K. (2018). Recent Developments in Solid Lipid Nanoparticle and Surface-Modified Solid Lipid Nanoparticle Delivery Systems for Oral Delivery of Phyto-Bioactive Compounds in Various Chronic Diseases. IJN.

[148] Luesakul U., Puthong S., Sansanaphongpricha K., Muangsin N. (2020). Quaternized Chitosan-Coated Nanoemulsions: A Novel Platform for Improving the Stability, Anti-Inflammatory, Anti-Cancer and Transdermal Properties of Plai Extract. Carbohydr. Polym..

[149] Al-Nemrawi N.K., Okour A.R., Dave R.H. (2018). Surface Modification of PLGA Nanoparticles Using Chitosan: Effect of Molecular Weight, Concentration, and Degree of Deacetylation. Adv. Polym. Technol..

[150] Hasan M., Messaoud G.B., Michaux F., Tamayol A., Kahn C.J.F., Belhaj N., Linder M., Arab-Tehrany E. (2016). Chitosan-Coated Liposomes Encapsulating Curcumin: Study of Lipid–Polysaccharide Interactions and Nanovesicle Behavior. RSC Adv..

[151] Almeida E.D.P., Santos Silva L.A., de Araujo G.R.S., Montalvão M.M., Matos S.S., da Cunha Gonsalves J.K.M., de Souza Nunes R., de Meneses C.T., Oliveira Araujo R.G., Sarmento V.H.V. (2022). Chitosan-Functionalized Nanostructured Lipid Carriers Containing Chloroaluminum Phthalocyanine for Photodynamic Therapy of Skin Cancer. Eur. J. Pharm. Biopharm..

[152] Alomrani A., Badran M., Harisa G.I., ALshehry M., Alhariri M., Alshamsan A., Alkholief M. (2019). The Use of Chitosan-Coated Flexible Liposomes as a Remarkable Carrier to Enhance the Antitumor Efficacy of 5-Fluorouracil against Colorectal Cancer. Saudi Pharm. J..

[153] Bian Y., Gao D., Liu Y., Li N., Zhang X., Zheng R., Wang Q., Luo L., Dai K. (2015). Preparation and Study on Anti-Tumor Effect of Chitosan-Coated Oleanolic Acid Liposomes. RSC Adv..

[154] Hasan M., Elkhoury K., Belhaj N., Kahn C., Tamayol A., Barberi-Heyob M., Arab-Tehrany E., Linder M. (2020). Growth-Inhibitory Effect of Chitosan-Coated Liposomes Encapsulating Curcumin on MCF-7 Breast Cancer Cells. Mar. Drugs.

[155] Dawoud M. (2021). Chitosan Coated Solid Lipid Nanoparticles as Promising Carriers for Docetaxel. J. Drug Deliv. Sci. Technol..

[156] Bhaskaran N.A., Jitta S.R., Salwa, Cheruku S., Kumar N., Kumar L. (2022). Orally Delivered Solid Lipid Nanoparticles of Irinotecan Coupled with Chitosan Surface Modification to Treat Colon Cancer: Preparation, in-Vitro and in-Vivo Evaluations. Int. J. Biol. Macromol..

[157] Wang J., Wang Y., Meng X. (2016). Chitosan Nanolayered Cisplatin-Loaded Lipid Nanoparticles for Enhanced Anticancer Efficacy in Cervical Cancer. Nanoscale Res. Lett..

[158] Truong T.H., Alcantara K.P., Bulatao B.P.I., Sorasitthiyanukarn F.N., Muangnoi C., Nalinratana N., Vajragupta O., Rojsitthisak P., Rojsitthisak P. (2022). Chitosan-Coated Nanostructured Lipid Carriers for Transdermal Delivery of Tetrahydrocurcumin for Breast Cancer Therapy. Carbohydr. Polym..

[159] Lu B., Lv X., Le Y. (2019). Chitosan-Modified PLGA Nanoparticles for Control-Released Drug Delivery. Polymers.

[160] Yang R., Yang S.-G., Shim W.-S., Cui F., Cheng G., Kim I.-W., Kim D.-D., Chung S.-J., Shim C.-K. (2009). Lung-Specific Delivery of Paclitaxel by Chitosan-Modified PLGA Nanoparticles via Transient Formation of Microaggregates. J. Pharm. Sci..

[161] Nag S., Das Saha K. (2021). Chitosan-Decorated PLGA-NPs Loaded with Tannic Acid/Vitamin E Mitigate Colon Cancer via the NF-ΚB/β-Cat/EMT Pathway. ACS Omega.

[162] Badran M.M., Alomrani A.H., Harisa G.I., Ashour A.E., Kumar A., Yassin A.E. (2018). Novel Docetaxel Chitosan-Coated PLGA/PCL Nanoparticles with Magnified Cytotoxicity and Bioavailability. Biomed. Pharmacother..

[163] Badran M.M., Mady M.M., Ghannam M.M., Shakeel F. (2017). Preparation and Characterization of Polymeric Nanoparticles Surface Modified with Chitosan for Target Treatment of Colorectal Cancer. Int. J. Biol. Macromol..

[164] Ahmad N., Ahmad R., Alam M.A., Ahmad F.J., Amir M., Pottoo F.H., Sarafroz M., Jafar M., Umar K. (2019). Daunorubicin Oral Bioavailability Enhancement by Surface Coated Natural Biodegradable Macromolecule Chitosan Based Polymeric Nanoparticles. Int. J. Biol. Macromol..

[165] Giacone D.V., Dartora V.F.M.C., de Matos J.K.R., Passos J.S., Miranda D.A.G., de Oliveira E.A., Silveira E.R., Costa-Lotufo L.V., Maria-Engler S.S., Lopes L.B. (2020). Effect of Nanoemulsion Modification with Chitosan and Sodium Alginate on the Topical Delivery and Efficacy of the Cytotoxic Agent Piplartine in 2D and 3D Skin Cancer Models. Int. J. Biol. Macromol..

[166] Fathy M.M., Mohamed F.S., Elbialy N., Elshemey W.M. (2018). Multifunctional Chitosan-Capped Gold Nanoparticles for Enhanced Cancer Chemo-Radiotherapy: An Invitro Study. Phys. Med..

[167] Farmanbar N., Mohseni S., Darroudi M. (2022). Green Synthesis of Chitosan-Coated Magnetic Nanoparticles for Drug Delivery of Oxaliplatin and Irinotecan against Colorectal Cancer Cells. Polym. Bull..

